# Polyphenols: From Classification to Therapeutic Potential and Bioavailability

**DOI:** 10.3390/foods13244131

**Published:** 2024-12-20

**Authors:** Daria Ciupei, Alexandru Colişar, Loredana Leopold, Andreea Stănilă, Zorița M. Diaconeasa

**Affiliations:** 1 Life Science Institute, University of Agricultural Sciences and Veterinary Medicine, Manastur 3-5, 400372 Cluj-Napoca, Romania; daria.ciupei@usamvcluj.ro; 2 Faculty of Forestry and Cadastre, University of Agricultural Sciences and Veterinary Medicine, Manastur 3-5, 400372 Cluj-Napoca, Romania; alexandru.colisar@usamvcluj.ro; 3 Faculty of Food Science and Technology, University of Agricultural Sciences and Veterinary Medicine, Manastur 3-5, 400372 Cluj-Napoca, Romania; loredana.leopold@usamvcluj.ro (L.L.); andreea.stanila@usamvcluj.ro (A.S.)

**Keywords:** polyphenols, bioavailability, metabolism, quercetin, chlorogenic acid

## Abstract

Though ubiquitous in nature, polyphenols gained scientific prominence only after the pioneering work of researchers like E. Fischer and K. Freudenberg, who demonstrated their potential beyond traditional applications, such as in the leather industry. Today, these bioactive compounds are recognized for their diverse therapeutic roles, including their use as adjuvants in cancer treatment, cancer prevention, and their anti-inflammatory and antioxidant properties. Additionally, polyphenols have demonstrated benefits in managing obesity, cardiovascular diseases, and neuromodulation. Their synthesis is influenced by environmental and genetic factors, with their concentrations varying based on the intensity of these variables, as well as the stage of ripening. This review provides a comprehensive overview of polyphenols, covering their classification, chemical structures, and bioavailability. The mechanisms influencing bioavailability, bioaccessibility, and bioactivity are explored in detail, alongside an introduction to their bioactive effects and associated metabolic pathways. Specific examples, such as the bioavailability of polyphenols in coffee and various types of onions, are analyzed. Despite their promising biological activities, a significant limitation of polyphenols lies in their inherently low oral bioavailability. However, their systemic circulation and the bioactive by-products formed during digestion present exciting opportunities for further research and application.

## 1. Introduction

In ancient times, people firmly believed that diet and exercise were the most important sources of well-being. Hippocrates, the father of medicine, said in his Greek oath: “I will use the diet that will benefit my patients to the best of my knowledge and belief, and I will do them neither harm nor injustice” [1]. Their eating behaviors and focus on health led to better and longer possible lives, while medication was administered second, only in times of need. BC mankind did not have the advance on research and medicine available nowadays to know exactly the benefits of foods and the power they possess on human health, but throughout the decades, these benefits have been proven through research. The significant abilities of secondary metabolites like polyphenols were first noticed in the leather industry under the name “vegetable tannins” [2], later called “vegetable polyphenols” by Emil Fischer and Karl Freudenberg, but were restrained by the lack of analytical tools [3], an issue we do not encounter at this moment in time. After multiple formulations and reformulations, the definitions it is presented as are known today: polyphenols are secondary metabolites that are formed in plants and are generally involved in protection from excessive exposure from UV radiation or different disease-causing microorganisms [4]. When plants are exposed to UV radiation, environmental factors, microbes, or stresses that occur during its life, it accretes a significantly higher number of phenols. There are studies that support the argument that plants grown in soil poor in nutrients and in higher temperatures accumulate a higher content in phenolic compounds. Polyphenols are phytochemicals that have health-benefiting potential, from being used in chronic disease or cancer treatment [5,6] to simply being used as preserving agents [7,8] or antioxidants [8]. They are the highest source of antioxidants in the nourishment of human species, favoring well-being. Although they are the largest known source of antioxidants for humans, their disadvantage is their low bioavailability and rapid absorption, which leads to excretion via urine [9]. The aim of this paper is to evaluate the bioavailability of two significant classes of polyphenols known for their substantial health benefits. Additionally, this study seeks to provide a comprehensive understanding of the importance of polyphenol consumption by highlighting their bioactivity and the health effects mediated by the metabolites formed during digestion.

## 2. Structure of Polyphenols: Classification

**Figure 1:**
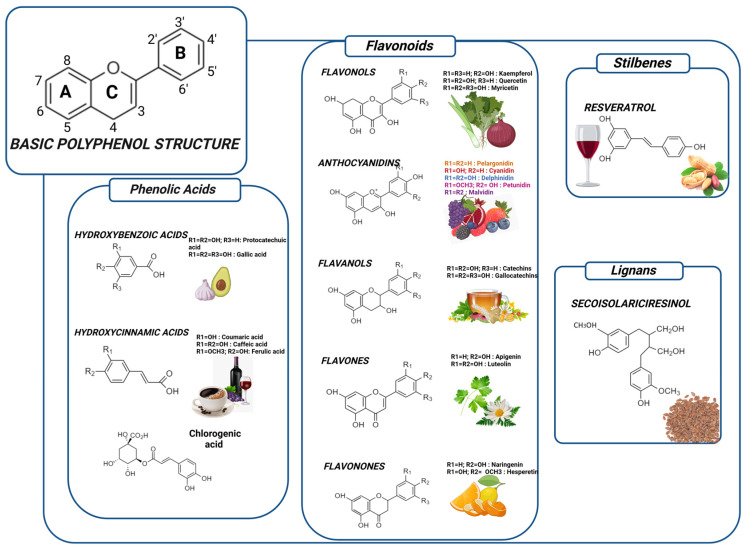
Polyphenol structures and representatives, along with some illustrations on where they could be found in plant-derived foods.

Polyphenols represent a diverse group of plant secondary metabolites with widespread occurrence in nature. Structurally, they are made of phenol units (Figure 1). More commonly, they exist in conjugated forms, with sugar residues linked to hydroxyl groups; direct links between sugar and aromatic carbon also exist. More than 8000 varieties of polyphenols have been identified in different plants throughout the years [10]. As they are considered the biggest class of phytochemicals, they ought to have another classification, dividing them into four most important subgroups: flavonoids, phenolic acids, stilbenes, and lignans, as presented in Figure 1.

### 2.1. Phenolic Acids (PA)

Phenolic acids can be defined as derivates of the benzoic acid or cinnamic acid. Hydroxycinnamic acids are more likely to be encountered in different plant species than hydroxybenzoic acids, and are predominately encountered in PA such as *p*-coumaric, caffeic, ferulic, and sinapic acids [11]. Mo often than not, they are found bound to an amine, ester, or glycoside. The literature highlights the great absorption of hydroxycinnamic as well as hydroxybenzoic acids, even though most information is known about hydroxycinnamic acids. Hydroxybenzoic acid (HBA) generally comes in small quantities, but it can be found in some red fruits, herbs, radishes, or onions, and it exists in some alcoholic beverages like wine or beer [12,13].

**Figure 2:**
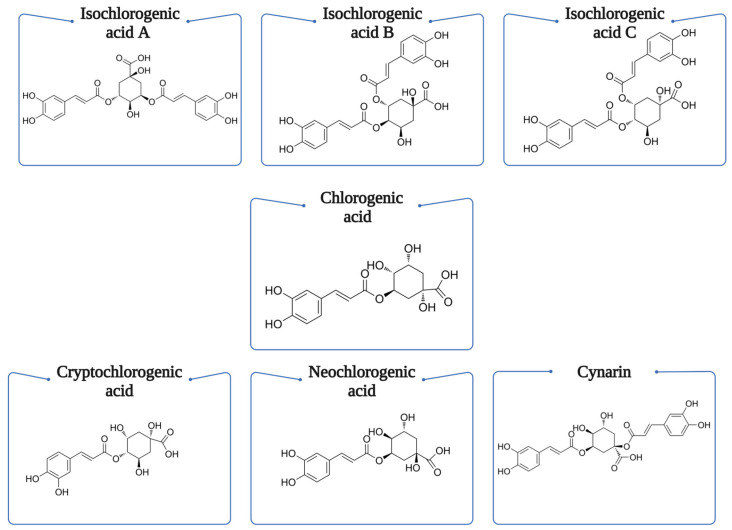
Chlorogenic acid and its isomers.

Hydroxycinnamic acids (HCAs) are merely found in their bound forms, rarely in their free one. Caffeic acid is the main compound, representing over 70% of the total hydroxycinnamic acid of most fruits, concentrations that decrease during the ripening. Ferulic acid is abundant in cereal, found in the aleurone layer and pericarp, averaging around 98% of PA [14]. HCAs are absorbed easily by the stomach and small intestine; afterwards, the conjugation process is catalyzed by detoxifying enzymes. Consumption of HCA has been associated with lowering rates of cardiovascular disease, skin cancer, Alzheimer’s disease, and other brain dysfunctions, as well as obesity and diabetes, when in vivo orally administrated to rodents [11,15]. Chlorogenic acids are abundant in fruit and vegetable species. The most representative sources of CGAs are coffee beans, potatoes, eggplants, and sunflower seeds. They exist displayed as four different isomers of caffeoylquinic acid, 1-CQA, 3-CQA, 4-CQA, 5-CQA (Figure 2). Coffee is considered to be the main source with the largest amount of CGAs, their concentration being 6–12%. Their properties were discovered in 1837 in the work conducted by Robiquet and Bourton, but the name “chlorogenic acid” was given by Payen in 1846. Later along the decades, isomers of CGAs were discovered, and given names by the IUPAC commission [16]. Table 1 describes HCA-rich foods and their importance on health management.

### 2.2. Flavonoids

Flavonoids are made of two aromatic rings linked by three carbon atoms forming an oxygenated heterocycle. Researchers over the decades have discovered over 4000 flavonoids. Their name derives from the latin “flavus”, signifying yellow or golden. Given their abundance, they have been divided into the following subclasses: flavonols, flavones, isoflavones, flavanones, flavanols, and anthocyanins. Most commonly, we encounter flavonol glycosides, between five and ten different compounds, mainly representatives of the group being quercetin and kaempferol. Flavanols were detected as both monomers (catechins) or polymers (proanthocyanidins). Epicatechins are remarkable in tea, being stable to heat exposure, while proanthocyanidins are known for formatting complexes with salivary proteins, and giving an intense and astringent taste that changes with ripening [28]. Anthocyanins are pigments found in the epiderma of the fruit, as vacuolar juice. They are responsible for the coloration of red fruits, berries, grapes, and many other fruits and vegetables.

Being the most studied of the polyphenols means not only basic information about their coloring properties and flavoring capacity is known, but also about their enzyme-blocking properties, used as a treatment for dementia by blocking the Acetylcholinesterase enzyme in the brain. The inhibition of enzymes (COX) for anti-inflammatory purposes has been studied, as well as steroid genesis modulating, countering antibiotic resistance, disease-combating activity, and combating neurodegenerative diseases [29,30]. Another important study underlines the possibility of using scavenging enzymes that detoxify cancerogenic cells, followed by their elimination. Flavonoids are claimed to be useful in inducing apoptosis, suppressing invasiveness and autophagy [31].

Recent mechanistic studies by Sitarek et al. (2024) have revealed new insights into flavonoids’ action as DNA topoisomerase inhibitors, demonstrating their potential through multiple-cell models. This work has identified novel pathways through which flavonoids exert their anticancer effects, particularly highlighting their role in DNA topology modulation [32]. Quercetin is a flavonol found in more than 20 plant species of fruits, vegetables, or grains. The main sources of flavonoids and their biological activities are shown in Table 2.

**Figure 3:**
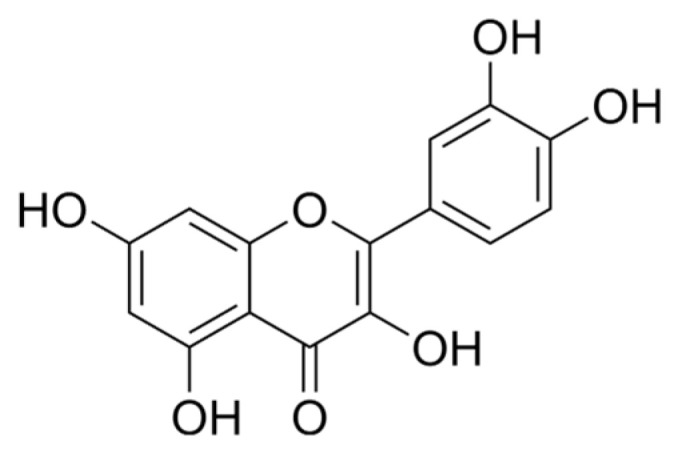
Quercetin according to IUPAC.

Quercetin and naringenin are two of the most well-researched and potent flavonoids, known for their wide range of health benefits. Quercetin, found in foods like apples, onions, and berries, is celebrated for its powerful antioxidant and anti-inflammatory properties, which help reduce the risk of chronic diseases like heart disease and diabetes [45]. Naringenin, primarily found in citrus fruits such as grapefruit, has similar antioxidant benefits and is also known for its ability to support metabolic health by improving insulin sensitivity and reducing inflammation. The selection of quercetin and naringenin for investigation or inclusion in health interventions is grounded in their substantial therapeutic potential, widespread presence in commonly consumed foods, and their demonstrated capacity to support cardiovascular health, regulate glycemic control, and mitigate oxidative stress. These properties position quercetin and naringenin as significant contributors to preventive healthcare strategies, offering promising avenues for the management of chronic conditions and the enhancement of overall wellness [46]. Quercetin is a flavonol found in more than 20 plant species of fruits, vegetables, or grains, and its chemical structure is presented in Figure 3.

The word “quercetin” derives from the Latin word “*Quercetum*”, meaning oak, or oak forest, not being able to be produced in the human body. It is yellow-colored and liposoluble—soluble in alcohol and insoluble in water depending on its temperature (being lightly soluble in hot water). The International Union of Pure and Applied Chemistry (IUPAC) gave quercetin the following name and chemical formula: 2-(3,4-dihydroxyphenyl)-3,5,7-trihydroxychromen-4-one and C_15_H_10_O_7_. It has been used for its properties for a long time, as listed in Table 3.

Naringenin is another valuable flavonoid compound, specifically, a primary flavanone. It has been reported in higher quantities in citrus fruits like oranges, grapefruit, and lemon or lime peels. Concentration depends on the harvesting method and environmental conditions. Naringenin is the aglycone of naringin. Naringin is the inactive form that will further be transformed into naringenin by gut bacteria. It is known for its antioxidant capacity and many other pharmaceutical benefits. The compound’s chemical formula is 5,7-dihydroxy-2-(4-hydroxyphenyl)-2,3-dihydrochromen-4-one, or C_15_H_12_O_5_. Lipophilic propriety gives the compound the characteristic of being soluble in organic solvents, such as polar alcohols (e.g., ethanol). Naringenin has been intensely studied for its neurological modulations against diseases and disorders [57]. An interesting aspect of naringenin found in tomatoes during ketchup making is its conversion from naringenin chalcone to naringenin [58]. The following provides an overview of naringenin-type flavonoids, including key compounds such as naringenin, naringin, hesperidin, and others, along with their primary sources and associated pharmacological activities (Table 4).

### 2.3. Stilbenes

**Figure 4:**
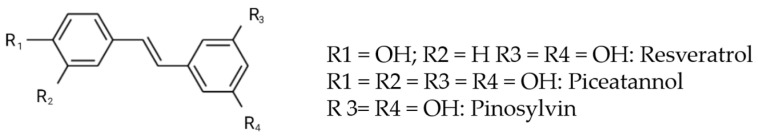
Structure of stilbenes.

These compounds can act as an antifungal, synthetized in cases of injury or infected tissue. Structurally, they have a C_6_–C_2_–C_6_ skeleton, most often presented with two isomeric forms (Figure 4). Stilbenes are biosynthesized in times of biotic and abiotic stress that occurs in the plant’s life, such as microbial attacks, extreme heat exposure, and oxidation [67]. A more intensely studied stilbene is resveratrol (3,4′,5-trihydroxystilbene), that naturally occurs in grapes [68]. Moreover, resveratrol acts as an antioxidant agent, fights cancer cells, improves lipid metabolism, and holds anti-aging and cardioprotective properties [69]. It is found abundantly in grapes, peanuts, wine, and berries. Resveratrol inhibits low-density lipoprotein (LDL) oxidation and therefore shows great importance in lowering rates of cardiovascular diseases. Its help in the obesity epidemic is linked to improving calorie restriction by stimulating Silent information regulator 2 (Sir2) [70,71].

### 2.4. Lignans

Lignans possess a unique chemical structure characterized by two phenylpropanoid units (C_6_-C_3_) connected at their β carbons (C_8_-C_8′_). Their basic skeleton consists of two coniferyl alcohol residues joined in a 2,3-dibenzylbutane structure. The diversity in lignan structures arises from different oxidation patterns and varying substitutions on the aromatic rings, typically including methoxy (-OCH_3_) and hydroxyl (-OH) groups [72]. The major dietary lignans can be classified based on their oxidation level and substitution patterns:

*Secoisolariciresinol diglucoside (SDG)* contains two glucose molecules attached to the main structure through ester bonds. Its aglycone form has two hydroxyl groups on each aromatic ring and two methoxy groups.

*Matairesinol* features a more complex structure with a lactone ring formed between the two phenylpropanoid units, containing two methoxy and two hydroxyl groups.

*Pinoresinol* has a unique furofuran structure formed by two tetrahydrofuran rings, with methoxy and hydroxyl substitutions on the aromatic rings.

*Lariciresinol* represents an intermediate oxidation state between pinoresinol and secoisolariciresinol, containing one tetrahydrofuran ring.

During microbial metabolism in the gut, these plant lignans undergo several biotransformation steps. The key process involves demethylation, dehydroxylation, and reduction reactions that convert them into enterolignans enterodiol (ED) and enterolactone (EL). This transformation significantly alters their chemical structure and biological activity compared to the parent compounds [73,74]. Recent research by Berenshtein et al. (2024) has provided additional insights into how these transformations are influenced by external factors, demonstrating that food processing and storage conditions significantly impact lignan stability and bioaccessibility. Their findings emphasize the importance of proper handling and processing methods in optimizing dietary lignan intake and maintaining their biological potential [74].

**Figure 5:**
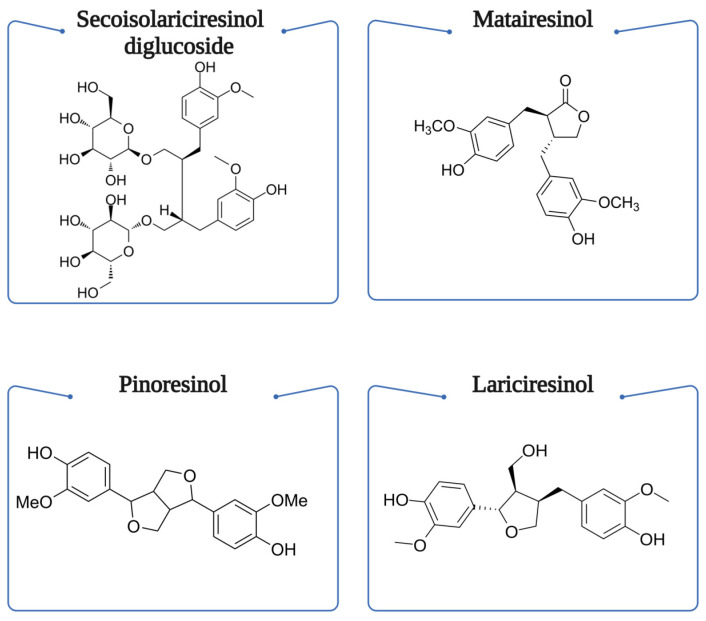
Structure of lignans.

They are considered phytoestrogens, as they can stimulate the secretion of certain hormones. Structurally, they are a linkage of a 2,3-dibenzylbutane resulted after the dimerization of two cinnamic acid residues [75]. Lignans are weakly estrogenic or anti-estrogenic, found mostly in seeds such as flaxseeds and sesame seeds, and less in legumes and other plant-derived foods. Their estrogenic mechanism and other bioactivity-linked mechanisms offer lignans potential in preventing heart diseases and other chronic diseases. Most commonly in foods, lignans such as lariciresinol, matairesinol, pinoresinol, and secoisolariciresinol have been identified. Flaxseeds and sesame seeds contain exceptionally high lignan concentrations (335 mg/100 g and 373 mg/100 g, respectively), exhibiting up to 100-fold higher content compared to other dietary sources. Following ingestion, these plant lignans undergo biotransformation by gut microbiota to produce enterolignans, their bioactive metabolites [76]. They are often found in human urine or plasma and are correlated with decreasing risks of coronary heart disease [77]. The microbial metabolism of lignans is particularly interesting, as studies have shown that their bioavailability and therapeutic effects are highly dependent on gut microbiota composition. Recent research indicates that specific bacterial strains, including *Bacteroides, Clostridium*, and *Eubacterium* species, are crucial for converting plant lignans into bioactive enterolignans. The efficiency of this conversion varies significantly among individuals, with factors such as diet, antibiotic use, and overall gut health influencing the process. Additionally, the presence of dietary fiber alongside lignans may enhance their biotransformation and absorption, as demonstrated in studies with whole grain and flaxseed consumption. The enterolignans ED and EL exhibit different biological activities compared to their parent compounds, with research suggesting they may have stronger antioxidant and anti-inflammatory properties. Moreover, their structural similarity to endogenous estrogens allows them to modulate estrogen-signaling pathways, contributing to their potential protective effects against hormone-dependent cancers and cardiovascular diseases [78,79,80]. Figure 5 shows the chemical structure of four more common lignans.

## 3. Dietary Intake of Polyphenols

Dietary intake of polyphenols varies from culture to culture, from one’s eating habits and preferences to another’s, making it extremely difficult to estimate the average daily intake. In 1976, researcher Kuhnau determined the total polyphenol daily intake in the United States to land approximately at 1 g/d [81], this value remaining the most used etalon for most researchers. Flavonol intake in the U.S., Denmark, and Holland is about 20–25 mg/d, while in Italy, it was estimated a mean, with 15 mg/d higher. Current dietary reference intakes for anthocyanins have not been established in most countries. China is the only nation that has proposed a specific intake level of 50 mg/day. According to NHANES 2007–2008 data, the estimated average intake in the United States for adults aged ≥ 20 years is 11.6 ± 1.1 mg/day, with women showing higher consumption (12.6 ± 1.5 mg/day) compared to men (10.5 ± 0.8 mg/day). Regarding safety, the Joint FAO/WHO Expert Committee on Food Additives has established an acceptable daily intake of 2.5 mg/kg body weight per day, specifically for anthocyanins derived from grape-skin extracts. Due to high coffee consumption, the hydroxycinnamic acid intake for several cups of coffee may result in ingesting > 500 mg/d [28,81].

### 3.1. Bioavailability

**Figure 6:**
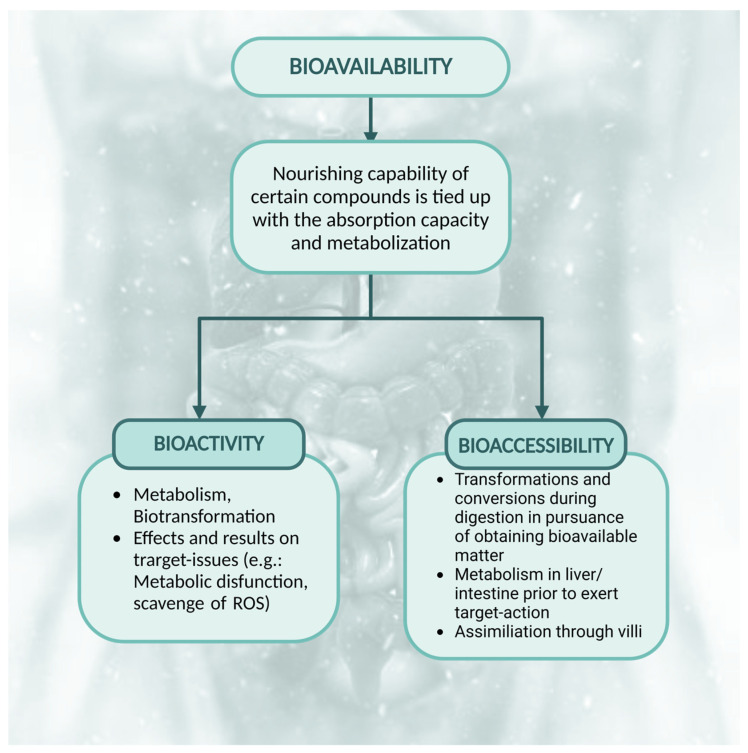
Bioavailability and term appliance.

Referring to bioavailability indicates the capacity of the substance/compound to be assimilated and metabolized by the host that ingested the supposedly bioavailable matter in question. Because bioavailability is tightly linked to the terms bioactivity and bioaccessibility, it is necessary to take into consideration the way the substance is transformed during transformations, conversions, and metabolization, the pathways it reaches, but also the effect it possesses on the host’s ailment (Figure 6).

Bioavailability of polyphenols can be affected by a number of factors, the main one being the interaction between other compounds in the food matrix, as well as their chemical structure. The impact that the food matrix can have on the accessibility of polyphenols and its absorption is highly dependent on the food matrix from where it is released [82]. This interconnection between their bioaccessibility and bioactivity will be affected positively or negatively by these intermolecular interplays. There are three main classes of macromolecules: carbohydrates, lipids, and proteins, each having their own particular way of interaction with polyphenols. Interaction with macromolecules is of great interest, being one of the main factors that can impact the effect polyphenols possess. The average human diet is most likely to be abundant in carbohydrates, with proteins and lipids following in second and third place, respectively. Since polyphenols have been recognized for their potency of being aids as immune system boosters, evaluation of their interaction with the main classes that characterize the human diet was emphasized thoroughly in the last century. Food matrixes’ interactions with phenolics can serve as carrier agents through the digestion, making them less prone to oxidation [83].

The interaction between carbohydrates and polyphenols is likely linked to their frail hydrophobic and hydrogen bonds. An important factor of this reaction refers to the phenolic structure; favorable compounds are flavonoids and catechins, unfavorable reactions were shown in the methoxylation of phenolic acids. One of the most abundant carbohydrates found in plants, starch, has been studied over the years in this scope. Interaction with polyphenols show gelatinization and retrogradation of starch when interacting with flavonoids; gelatinization is increased in quercetin and green tea flavan-3-ol, while retrogradation is repressed [82]. Other functions of starch are affected, such as thermal, physiochemical, or digestibility. Main changes that affect digestibility rate are starch structure, granule surface smoothness, relative crystallinity, and short range molecular order [83]. If it were to give the simplest definition of what starch is, it would have to be that it represents the stored energy of different plants reserved as feed when other resources are not available. Chemically, it is a polymer made out of linear and branched microstructures linked through α-d-(1-4) glucose bonds. The linear structure is called amylose, with α-1,4 glucose bonds. The branched one is called amylopectin, characterized by its α-1,4 chain bonded by α-1,6 glucose links. The amylose/amylopectin proportion is influenced by its source and age, but also, its evolution is dependent on the survival needs of the plant in question, so it can highly differ from region to region [84]. Interplay of polyphenols and starch is concluded to be due to the hydroxyl-group-containing polyphenols and non-covalent bonds of the polymer discussed (hydrogen bonds and electrostatic and ionic interactions). End products can be V-type inclusions with hydrophobic contacts or non-inclusion with weaker binding forces. Notably, V-type inclusion can restrict hydrolysis of the starch molecule.

Furthermore, phenols can also bind directly to the target enzyme, decreasing its activity and, therefore, the starch’s digestibility. This aspect is notable in tea polyphenols, flavonoids, ferulic acid, quercetin, gallic acid, tannic acid, *p*-coumaric acid, or sinnapic acid. Another mechanism of action is amylase, which lowers the activity during hydrolysis. In the case of excessive supplementation of polyphenols, it may enhance digestibility as a result of high complexation with a looser structure [83]. In the case of flavonoids interacting with different starch-rich foods, Shanshan Gao et al., while studying quercetin and starch from Tartary buckwheat, managed to highlight the bonding through non-covalent linkages during the process of gelatinization, resulting in a V-type structure [85]. Libo Wang et al. showed the diminished digestibility of quercetin–starch (Tartary buckwheat) complexes by modifying the polymer’s structure and limiting the specific enzyme activity. Compared to rutin, quercetin had a stronger effect [86]. Interaction of tea polyphenols with wheat starch showed a reducing rate of retrogradation in their interaction. Studies show the interaction of these two compounds to be through hydrogen links, having almost an identical effect to quercetin without forming the V-type, resulting in an increasing content of resistant starch and reducing digestibility [83,87]. Due to these interactions, there could potentially be a loss in polyphenol content.

For non-starch compounds, such as fiber-rich foods, interaction of polyphenols might act as carrier agents for these bioactive compounds [82]. Dietary fibers are categorized as carbohydrate polymers susceptible of low digestibility in the upper-digestive path of the digestive system, but it can be highly transformed and metabolized in the large intestine. The literature shows that dietary fiber has the capacity of modulating the microbiome, but can also aid in the colonization of the intestinal microbiota. The final products of fermentation in the colon are short-chain fatty acids, gas, and water [88]. Commonly, the linkage is formed through non-covalent bonds, including hydrogen bonds, van der Waals forces, and hydrophobic interplays. Van der Waals forces occur through the existence of functional groups that are capable of creating polarized molecules; in the case in which hydrogen bonds are previously formed, it results in a shorter distance, permitting van der Waals forces to take effect. Insoluble polyphenols and dietary fiber have the capacity to aggregate [88,89]. Of great impact on the bondage are ionic strength, temperature, or pH level. Due to a high ionic strength, the association of molecules is increased, leading to a higher probability of formation of hydrophobic interactions, since hydrophobic molecules aggregate together to minimize exposure to the ionic force. In the case of hydrogen bonds, exothermal reaction is needed, and the opposite, endothermal reaction, for hydrophobic ones. With a higher temperature that results in the association of molecules, hydrophobic bonds have a more plausible forming capacity, while in H bonds, when the temperature increases, the association decreases, and thus, so does the formation of the link. The increase in size of the polyphenols’ molecules can positively affect the degree of the bonding. The solubility of the phenol is also important: the hydrophobic character of fiber is complexed by hydrophobic polyphenols and likewise in the case of hydrophilic polyphenols. The carrier agent effects of polyphenol–fiber complexes throughout the digestive tract are remarked: it is shown that they possess a low bioavailability in the small intestine, but it increases in the colon, having a higher dose released [90,91].

A decrease in particle size can show positive results in the availability in the upper digestive tract. In the lower digestive tract, polyphenol–fibers are released (seemingly in a higher dose due to the carrier-like capacity of the fiber) and the complexes undergo the process of fermentation and transformation in catabolites that are bioaccessible [88]. Short-chain fatty acids are a result of the fermentation process of dietary fibers, and their increase might be a result of linkage with the phenol group [88,90].

Proteins mostly interact through non-covalent bonding and hydrophobic bonds, followed by a stabilization by hydrogen bonds. In the case of oxidized flavonoids, the compounds formed, quinones, can form irreversible covalent bonds. Interplays of these two classes of compounds can be specific or non-specific. Specific interaction refers mostly to enzymes/proteins with a strong tertiary structure (immunoglobulin in milk, myoglobin in muscle). Larger polyphenols have a better binding capacity; molecular weight is taken into account when discussing this type of interaction [82]. Other factors to take into account are temperature, ionic strength, and pH. In the case of pH, it is observed that pH levels for binding in tea polyphenols range from 3.6 to 8. Biological effects are often seen in the case of interaction between phenols and proteins; a significant one is the astringency, as a consequence of salivary proteins and polyphenols in red wine, tea, or coffee, causing the preserving of dry mouth and a tightening sensation. The interaction with salivary proteins is mostly encountered in tannins [92,93]. In the case of tea, with addition of milk, it results in a lower astringency feeling, binding the flavan-3-ol to the caseins and whey proteins. A considerable aspect is the functionality of proteins: studies show that the binding of these molecules with polyphenols can result in blocking their amino acid functions, thus decreasing their bioavailability. Polyphenol’s bioavailability can increase through linking with proteins. Stabilization, in this case, means enhancing the antioxidant capacity, preventing the polyphenols from oxidation. Reports show that milk proteins and catechins from tea can bind and better the intestinal transportation and absorption of the tea polyphenols. In animal studies, soy proteins and polyphenols showed an increase in bioavailability and transport throughout the GI tract from these forms of complexes [82].

Interaction with lipids has been highlighted in the case of plant oils. It was demonstrated that some lipids could increase acute absorption of flavonoids, like quercetin. Green tea catechins can serve as food additives to fatty products that are easily oxidized. As a downside, tea catechins can affect the pH and microstructure of milk products such as cheese, causing changes in organoleptic characteristics and hardness. It has been noted that polyphenols can influence triglyceride and fatty-acid synthesizing, increasing levels in rat models when incubation of resveratrol and hepatic cells was conducted, but also decreasing enzyme activity of acetyl-coA carboxylase. One mechanism of action that authors have noted throughout their research along the years is the lowered or later absorption of lipids in the gut as a result of the inhibition of pancreatic lipase (that breaks down lipids). Polyphenols can also incorporate in the lipid layer, grow in size, and modify the physiological and chemical characteristics of the fat droplets. Also, a possible way of stabilizing the polyphenols could be taken into account—incorporating them into lipid particles, making them less prone to degradation in the GI tract, but also the possibility of the formation of liposomes, that might be a possibility of slow and controlled release of the polyphenols [82].

### 3.2. Bioavailability of Phenolic Compounds

#### 3.2.1. Phenolic Acids

Phenolic acid sources vary, as they are commonly found in all food groups; higher concentration has been reported in cereal, legumes, oilseeds, beverages, and herbs [94]. They contribute to nutritional and organoleptic properties, but they are significantly used for their health contribution, as they are powerful antioxidants [11,95]. Bioavailability of these compounds depends on their free form, level of conjugation, or different treatments they might undergo. In the processing of cereal, preliminary methods can increase the amount of free form PAs. Even though milling, air-classification, and dehulling can either increase the bound-forms of PAs or decrease them overall, further bioprocessing like germination, fermentation, or enzyme treatments can impact the increase in free form phenolics. In dough, the fermentation process aids in destroying the cell wall, as most phenolic acids are esterified to it. The further absorption by the host through GI barrier, entering the bloodstream, could be the perceiving of the absorbed phenolics as xenobiotics and the body’s response of removing these substances. HCAs suffer transformations under the processes of glucuronidation, sulfation, and further oxidation to benzoic acid (and derivates) that are glycinated into hippuric acid derivates. These transformations enhance elimination through phase I or II. One of the major enzymes responsible for the metabolization of xenobiotics is cytochrome P450 monooxygenase. Debrisoquine hydroxylase and mephenytoin hydroxylase depend on the gene expression variability of the host. Acetylation enzymes are dependent on the phenotype that is influenced by the territory in which the host is living in. Slower acetylator phenotypes can have a decreased activity in the liver’s NAT1 and NAT2 genes, essential in the transformation of acetate into acetyl-CoA [95]. Thermo liability has been proved in a study conducted by Yu and Beta, showing an increase in free ferulic acid and *p*-hydroxybenzoic acids during baking [96]. Because free form is less common, bound forms of dietary fiber compounds reach the intestinal microbiota and are, therefore, degraded by it. Hydrolysis by microbial esterase is the proposed pathway, conducting in the release of conjugated/bound form and liberating the free form of HCAs in vivo [95]. A study conducted in 2021 by Wenfei Tiai et al. regarding phenolic acids’ in vitro digestion of whole wheat products showed an increase in the first hour of digestion. Trans-ferulic acid increased throughout the Gastric phase (44.82 to 63.36 µg/g), but also significantly though the Intestinal phase (85.10 µg/g). Similarly, 4-hydroxybenzoic acid, vanillic acid, and sinapic acid showed an increased activity. An important mention from this study is the fact that digestive enzymes and dramatically reduced pH were catalysts for the releasing of PA. Alkaline conditions may help break the insoluble bond, but not in all cases. In the case of vanillic acid, because of the entrapment in the fiber–protein matrix, these conditions might not help releasing the insoluble form. Colonic fermentation of probiotic strains with the whole wheat products show production of PAs from the digested residues [97].

#### 3.2.2. Flavonoids

Flavonoids are commonly found in teas, citrus fruits, berries, and leafy vegetables responsible for exerting health benefits upon the host [98]. Even though some of them are easily found in food, others are solely found in a certain group; for example, isoflavones are only found in legumes. The low bioavailability is caused mainly by phase II metabolism. In most cases, they suffer from sulfation, methylation, and glucuronidation in the upper part of the GI tract and liver and conjugated forms exist in plasma after flavonoids are ingested. A critical factor for the bioavailability of flavonoids is represented by their molecular weight: the bigger the weight, the harder the absorption. Polymeric proanthocyanidins have a large molecular weight, making it an inconvenience in their metabolization in the stomach or small intestine, passing right through, reaching the colon and getting catabolized by the microbiota. The produced microbial metabolites could be subjected to entering the bloodstream and urinal excretion. Variation in means of absorption can differ even on the same compound within conjugates. In the case of glycosides, it might vary; reports show that bioavailability of apple quercetin glycosides was 30% of that from onions. Sugar linkage can also affect the rate of absorption of flavonoids [99]. Quercetin glucosides are absorbed faster and the plasma concentration peak was significantly higher than its rutinosides in humans. Glucosides are supposedly in the small intestine, whilst rutinosides are absorbed in the colon, post deglycosylation [99,100]. Another important factor is metabolic conversion. For quercetin, encountered in plasma were sulfate or methyl conjugates, but not its aglycone or glycosides. Also, in the case of cathechins, only conjugates were found in plasma. Colonic metabolization heavily impacts polyphenols, as a tremendous amount of polyphenols do not get metabolized in the stomach or small intestine, reaching the colon intact [99]. The degradation of the bacteria present in the gut results in simple phenolic acids and absorption in the bloodstream [101]. Proanthocyanidins were known to be catabolized to phenylacetic acid, mono- and dihydroxyphenylacetic acids, mono- and dihydroxyphenylpropionic acids, and hydroxybenzoic acid; anthocyanins were catabolized mainly to protocatechuic acid [102].

#### 3.2.3. Stilbenes

Stilbenes are polyphenols; their production de novo is caused as a mechanism of protection in situations of stress, such as fungal infections, toxins or to inhibit bacterial growth [103]. They are known under the name of allelochemicals [104] or phytoalexins [103]. Sources are usually represented by grapes (skin), peanuts, rhubarb, blueberries, or raspberries [103,104]. Most representative for this group is resveratrol; this compound can be found in two conformations, predominantly being in the *trans-* form (found in red grapes and peanuts, naturally). The cis- form of resveratrol can be encountered in wine. Moreover, the *cis-* form molecule might present a decreased bioactivity compared to the *trans-* due to its isomerization [103]. The absorption of resveratrol is high, but its bioavailability is poor, being easily concluded in phase II conjugation. After ingestion of resveratrol, conjugates are present in serum within 30–60 min [105]. Circadian rhythm and type of meal (highly lipidic foods, bondage to proteins) can influence bioavailability [103]. It was concluded that the best time for administering resveratrol was in the morning, being careful of not exceeding dosage, as it might be harmful, causing digestive issues, headaches, and nausea [103,106]. The dosage recommended is 100–1000 mg/day [103].

Stilbenes suffer different transformations after ingestion, like oxidation, reduction, conjunction mostly in the liver. For resveratrol, post oral ingestion, 70% is absorbed but only a small amount is available (5 ng/mL) with a 9 h half-life in plasma. Extensive hepatic conversions are responsible for the formation of sulfo- and glucorono-conjugates. Through phase II conjugation, compounds formed are hydrosoluble and easily eliminated [104]. Only 2% of resveratrol conjugates are detected in plasma and urine post-ingestion; major compounds found in urine are monoglucuronides, dihydroresveratrol monoglucuronide, resveratrol monosulphate, and dihydroresveratrol sulphate, according to Walle T. et al. [103,105]. When resveratrol passes through the upper part of the GI tract and arrives in the colon, about 75% can enter the coloncytes, metabolization by microbiome occurs, and transformation in metabolites such as dihydroresveratrol or lunularin can take place [103]. Pterostilbenes are also rapidly absorbed, with a faster rate compared to resveratrol, with four times the bioavailability. The methoxy groups attached to it give it an increased lipophilicity characteristic. Half-time rises up to 104 min. Its stability and bioavailability is higher due to lack of hydroxyl groups available for sulfation, owning only one [104]. Oligomerization and its degree also affects the bioavailability of these polyphenols, as a higher level determines a lowered bioavailability [107].

#### 3.2.4. Lignans

Lignans are phytoestrogens with a steroid-like structure, found in various seeds, grains, fruits, or vegetables in relatively low concentration, with importance due to a higher concentration being flaxseeds and sesame seeds [77,108]. Secoisolariciresinol has glycosidic bonds that are hard to hydrolyze, being unsusceptible of undergoing hydrolysis when passing through the upper part of the GI tract. Its diglucoside release, whatsoever, gets released in this portion depending on the food matrix it is linked with; deglycosilation of secoisolariciresinol could occur thanks to the brush border enzymes, in vivo study shows exitance of secoisolariciresinol in biofluids after high intake of SDG [108]. Significant for these compounds are the conversions occurring in the colon. Metabolization by intestinal microbiome leads to transformation in enterolignans, respectively enterodiol (ED) and entrolactone (EL) (also known under the name of mammalian lignans) [77,108]. The half-life of these compounds is between 5–13 h, remaining able to be detected even 8–10 h post ingestion. The cell density of entero-ligan-producing bacteria strains is more pronounced in women rather than in men, depending on their menstrual cycle (mid-luteal phase) and early pregnancy [109]. The bacterial metabolization of secoisolariciresinol can be a model for most common plant lignans. Luminal secoisolariciresinol can be sourced from the microbial transformation of plant precursors, or secoisolariciresinol diglucoside that was not absorbed. SDG is subjected to two consecutive deglycosilations and the end product is secoisolariciresinol monoglycoside (SDG). Demethylation of secoisolariciresinol results in demethyl-SECO, followed by transformation through demethylation and dihydroxylation into demethyl-dehydroxy-SECO, didemethyl-SECO (dihydroxy-ED), didemethyl-dehydroxy-SECO (hydroxy-ED), and ED. The oxidation of ED leads to EL. Obtaining EL can be described through mechanisms such as the generation of MAT after the dehydrogenation of SECO [108]. Another factor that might impact the bioavailability of lignans is the matrix of interlinkage, as positive results are obtained in patients with a diet rich in fiber [77]. The absorption of enterolignan after their production by bacteria in the large intestine is efficient; EL-sulfate, EL-glucuronide and ED-glucuronide were successfully detected after exposure of colonic cells to enterolignans. Conjugation and excretion happened within an 8 h window, remarkable being the fact that EL was eliminated with a faster rate than ED, as observed in in vitro studies. In one in vivo study conducted on blood samples from 27 female subjects, Adlercreutz et al. manage to show that the biologically active fraction of ED and EL, mainly being represented by their free, mono- and di-sulfated forms, adds up to 21–25% of the total (conjugated and non-conjugated) enterolignans. A total of 80% were biologically inactive [109,110].

### 3.3. Bioactivity of Polyphenols

Polyphenols exert their bioactivity primarily through their interaction with biological systems at the molecular level, often mediated by their metabolites formed during digestion. They play a critical role in scavenging reactive oxygen species (ROS) and chelating metal ions, thereby reducing oxidative stress, a key factor in the pathogenesis of various chronic diseases. Specific pathways, such as the inhibition of lipid peroxidation and upregulation of endogenous antioxidant enzymes (e.g., superoxide dismutase and catalase), underscore their antioxidant properties.

These general mechanisms are well illustrated in specific plant sources. A 2024 review by Olas has expanded our understanding of Asparagus officinalis polyphenols, demonstrating their multifaceted health benefits, including antioxidant, anti-inflammatory, and anticancer properties. This work provides new evidence for the bioactivity of asparagus-derived compounds and their potential therapeutic applications through multiple molecular pathways, particularly highlighting their role in oxidative stress reduction and inflammatory response modulation [40]. Additionally, polyphenols influence inflammation by modulating cytokine production, suppressing proinflammatory mediators such as interleukin-6 (IL-6) and tumor necrosis factor-alpha (TNF-α), and inhibiting the nuclear factor kappa-light-chain-enhancer of activated B cells (NF-κB) pathway. Their anticancer effects include inducing apoptosis through caspase activation, inhibiting angiogenesis by targeting vascular endothelial growth factor (VEGF), and suppressing metastasis via the modulation of matrix metalloproteinases (MMPs).

Cardiometabolic benefits are also significant, with polyphenols shown to improve lipid profiles, enhance insulin sensitivity, and regulate blood pressure through their interaction with nitric oxide (NO) pathways. Emerging research highlights their neuroprotective effects, including the modulation of neuroinflammatory processes and promotion of synaptic plasticity, making them promising agents for mitigating neurodegenerative diseases. Moreover, the interaction of polyphenols with gut microbiota contributes to their bioactivity, as microbial fermentation produces bioactive metabolites that influence systemic health.

These multifaceted bioactivities emphasize the importance of understanding not just the chemistry of polyphenols but also their dynamic interactions within the human body to fully harness their therapeutic potential.

#### 3.3.1. Cardiovascular Protection

Cardiovascular Diseases (CVD) involve the circulatory system’s malfunction. CVD include several diseases, including coronary artery disease, stroke, heart failure, or high blood pressure. Consumption of polyphenols is reported to reduce and prevent the risk of developing or aggravating the symptoms of the preexistent disease [111].

Resveratrol can be most significantly found in grapes, specifically in their skin. Other sources for resveratrol can be peanuts, berries, or medicinal plants [112,113]. Since resveratrol is found in higher concentration in grape skin, red wine can be considered abundant in this polyphenol. Low mortality rate as a cause of CVD in France has been associated with the consumption of red wine. This phenomenon has been named “The French Paradox” due to their high-fat diet, but with a low incidence of deaths caused by coronary heart disease. Explanation of this paradoxal event suggests that moderate consumption of wine can provide anti-inflammatory effects, decrease platelet aggregation, increase high-density lipoprotein and decrease low-density lipoprotein, as these are causes of developing the disease. Resveratrol fights proinflammatory cytokines through its mechanisms of action through the decreasing of NLR family pyrin domain that consists of three inflammasome routes, therefore decreasing levels of interleukin (IL)-1β production and gene expression. The mechanism of action for resveratrol involves action upon molecular sites to stimulate endothelial production of NO, reduction of oxidative stress, inhibition of vascular inflammation, and prevention of platelet aggregation. Studies on resveratrol’s antioxidant potency suggest that it inhibits nicotidamide adenine dinucleotide phosphate oxidase and further limits the production of ROS [112]. Studies show that resveratrol consumption is connected to lowering the expression of inflammatory markers (e.g., intercellular adhesion molecules and interleukin-8) in endothelial cells and lower diastolic blood pressure in systemic circulation, these two being causes of ischemic stroke and hypertension [114]. Resveratrol upregulates endothelial nitric oxide synthase expression followed by NO production from endothelial cells [115]. The inhibition of platelet aggregation begins through cellular signaling through the inhibition of p38 mitogen-activated protein kinase pathway, activating the NO production/cyclic guanoside monophosphate with resulting phospholipase C and protein kinase C inhibition, decreasing intracellular calcium concentration or ROS formation. As an anti-inflammatory agent, it acts similarly to aspirin: target of cyclooxygenase-1 and 2, hindering prostaglandin activity. Sirtuin-1 can be another route for obtaining the anti-inflammatory effects given by resveratrol [112]. SIRT-1 is an NAD+ dependent protein deacetylase that regulates aging, transcription, proliferation, apoptosis, and inflammation. Loss of SIRT-1 leads to vascular and cardiac aging. Activation of SIRT-1 through resveratrol implies modulation of enzyme activity, protein phosphorylation, and transcription factor function, deriving its properties in regard to modulation of the cardiovascular system. The post-transitional deacetylation of lysine residues by means of resveratrol-SIRT-1 activation increases the activity of endothelial nitric oxide synthase. Superoxide anions are scavenged by NO; the duality of these two mechanisms provide balance for endothelial health, and besides its malfunctioning being associated as an early sign of hypertension, it is responsible for upping the rate of vascular tone and prompting vascular remodel [114,116]. AMP- activated protein kinase (AMPK) is a cellular-signaling mechanism for regulating energy and cellular pathways, being activated in periods of metabolic stress, such as depletion of nutrients, hypoxia, and intoxications [117]. The indirect modulation of AMPK conducted by the ingestion of resveratrol can be explained by the SIRT-1 pathway activated by the phenolic compound, and the deacetylation of LKB1 serine–threonine kinase that directly phosphorylates and stimulates inactive AMPK. Activating AMPK provides antioxidant and anti-inflammatory characteristics, for a well-functioning and balanced in-cell environment of the CVS [114,118].

Resveratrol at 10–100 µM provides cardiac safety insulin resistance syndrome (metabolic syndrome) in human aortic endothelial cells and salvianolic acid A (natural polyphenol used in Chinese medicine) shows benefits for CVD protection against lipotoxicity-induced myocardial damage at >5µM, as presented in some in vitro studies [119].

As for other human studies, an impressing study conducted by Rafael Moreno-Luna et al., shows the reduction of diastolic blood pressure (BP) after treatment with polyphenol-rich olive oil [120]. Changes in lipid metabolism in adults with CVD are potent in a study prescribing a polyphenolic-abundant drink of cranberries [111]. The consumption of polyphenols gave a positive response in lowering inflammatory risk factors such as human C-reactive (CRP) and fibrinogen protein destruction, mitigating atherosclerosis and, ultimately, CVDs [121]. Flavonoids such as quercetin and kaempferol are supportive of cardiac cell function in mitochondria. ROS species degenerate this function by ischemia-reperfusion damage [122].

#### 3.3.2. Neuroprotection

Neurodegenerative diseases that appear in older populations as a consequence of aging is characterized by loss of brain function. Brain function is degraded when neurons and neutral stem cells are destroyed, modulated by a cellular and molecular system: glutamate excitotoxicity, oxidative stress, and abnormal apoptosis; other than genetic and environmental influences [123,124]. Polyphenols enhance detoxification and antioxidation enzyme activity as well as regulation of reactive oxygen activity. They also inhibit glutamate-induced apoptosis [119]. In the case of hereditary chronic diseases such as Parkinson’s disease, Alzheimer’s disease, Huntington’s disease and dementia, inflammation and oxidative stress play a huge role in further degenerating the brain’s function [124]. In human trials, studies show dietary α-linolenic acid’s intake as successful on a lower education and lower background group. Inhibition of these problems may aid in brain health, ease the symptoms, and ultimately make the patient’s life easier. The consumption of resveratrol, curcumin, quercetin, or catechins shows cognitive improvement, inhibits apoptosis (and therefore limits the destruction of dopaminergic cells), and promotes antioxidant activity. Administering quercetin in human subjects improved cells’ life and antioxidant activity [111,124]. Because of the attention the gut–brain axis has been given in the recent years in the field of neurological studies, it is important to mention that gut microbiota can modulate activity in the brain, and therefore, can be a great influence on neurodegenerative diseases such as the ones previously mentioned. A healthy gut microbiota can influence one’s being, from physical to mental health. The article “Polyphenols-gut microbiota interplay and brain neuromodulation” by S. Filosa et al. mentions the role of polyphenols as prebiotics, a critical aspect for an optimal microflora. The enzymatic transformation of microorganisms from the gut improves bioavailability of polyphenols; subsequently, these key secondary metabolites will be modulating the microbiome community, avoiding the risk of pathogenic microbes [125]. Only recently, scientists have begun to take in consideration not only the administration of probiotics and probiotic-rich foods, but also the importance of including prebiotics into this class of psychobiotics. A remarkable and well-recognized study was conducted by Aurelijus Burokas and collaborators, studying the administration and the beneficial effect of administering prebiotics in mice. The results are as follows: “reduced chronic stress-induced elevations in corticosterone and proinflammatory cytokine levels and depression-like and anxiety-like behavior in addition to normalizing the effects of stress on the microbiota” [126]. Different studies show that different bacteria strains can produce doses and different types of neurotransmitters that are secreted by the brain: *Escherichia and Enterococcus* spp.*- serotonin and Lactobacillus and Bifidobacterium*- γ-aminobutyric acid (GABA) [124].

Neuromodulation through the gut–brain axis and administration of polyphenols has been proven along the years through research, and due to the fact that most polyphenols are not absorbed, they get metabolized by the microbiome [127]. Through the ingestion and metabolization of polyphenols by intestinal bacteria existing in the gut, they improve the diversity of the microbiota, production of important metabolites such as SCFAs, assist in the production of different hormones and neurotransmitters, and also play an important role in contributing to neurodegenerative disease treatment [128]. SCFA production has been linked to neuroactive attributes, and its production is linked to the microorganisms’ influence on CNS via the vagus nerve. In the colon, SCFA production mainly include its acetate, propionate, and butyrate form through the fermentation of different substrates like fiber or resistant starch [129,130]. The importance of the vagus nerve has been underlined to aid in satiety, stress, and mood; therefore, bacteria in the gut interacting to activate it can have an impact on CNS. After their production through fermentation, they are absorbed by colonocytes via $H^{+}$-dependent or sodium-dependent monocarboxylate transporters. SCFAs do not enter through colonocytes but get transported to hepatocytes via portal circulation and used as an energy source. SCFAs bind G-protein-coupled receptors (GPRs), particularly the intensely studied GPR43 and GPR41, GPR109a/HCAR2 (hydrocarboxylic acid receptor) and GPR164, expressed in various cells across the GI mucosa to the immune and nervous systems. The cell of expression will determine the effects of activation of these different types of receptors. Regulating systemic functions via histone deacetylase activity promotes the acetylation of lysine residues from nucleosomal histones; this particular signaling mechanism has been linked to modulating the peripheral NS and CNS [130]. The SCFA–Microglia interlink has been studied; microglial maturation and function can be modulated through microbiota production of SCFA acetate, butyrate, and propionate, as they could determine it through the activation of FFAR2 (free fatty acid receptor-2) in FFAR2-deficient mice [131]. Glial and microglial cells are responsible for the elimination of unnecessary synaptic connections, necessary for the maturation and refining of the circuits and connections in the NS. In the same interaction between microglial cells and SCFAs, their acetate form provides information on reducing inflammation through IL-1b, IL-6, and TNF-a expression and p38 MAPK, JNK, and NF-kB phosphorylation. SCFAs can modulate neurotrophic factors, that are factors for regulating growth, survival, and to differentiate neurons and synapses in the CNS, supposedly through GPR41 or GPR43 receptors. SCFAs have a positive effect on different mental disorders by improving the activity of the brain through its discussed mechanisms, and therefore, being a great tool in their treatment. Successful data conclude their help in autism spectrum disorders, balancing the microbes in the colon, usually a common trait found in individuals suffering from autism disorder. The same goes for mood disorders; in depressed patients it was found that commonly, proinflammatory cytokines were present and patients suffering from depression had lower SCFAs in stool than non-depressed patients. The current literature shows that butyrate can act as an antidepressant, creating changes in energy levels, cognitive functioning and sociability inadaptation [130]. Quercetin-3-O-glucuronide is one of the most abundant metabolites of quercetin in vivo. Its administration might restore dysbiosis of the microbiome by restoring normal levels of bacteria in the colon, as an abnormal composition could lead to neuroinflammation. Quercetin-3-O-glucuronide has the ability to restore Aβ43 (amyloid β-43- characteristic for Alzheimer’s disease) and induce low levels of SCFAs, being associated with changes in the gut microbiome [128]. Anthocyanidins are also powerful in exerting neuroinflammatory properties with effects on the CNS as a result of modulation of the gut microbiota. Kynurenic acid resulting from metabolizing of the phenols generated from tryptophan metabolism could be used as a therapeutic tool for psychiatric diseases for its antagonist properties in regards to excitatory Aa receptors [132].

#### 3.3.3. Antioxidant and Anti-Inflammatory Effects

Imbalance in free radicals in the body leads to oxidative stress. As a defense mechanism, the body releases cells that might come from enzymes, bacterial cells, mammalian cells, and polyphenols. In oxidative stress, the mechanism of cell damage occurs due to the different chemical actions of oxygen-based free radicals. Scavenging of free radicals, preventing oxidation of lipids, and inhibiting the secondary products of oxygen reactions—hydrogen peroxides produced by NADPH oxidases and xanthine oxidase—are one the main characteristics of the antioxidant properties of polyphenols [119,121]. The advantage of hydroxyl groups present in polyphenols gives them the propriety to scavenge free radicals and chelate ions. Their nature allows them to be providers of electrons or hydrogen for reactive oxygen species (ROS). For chelation of metal ions, they act by giving the peroxide compound or metal oxides stability. Increasing the activity of certain enzymes, polyphenols restore a balance of the enzymatic redox system, an oxidative defense mechanism, averting inflammation. Although they play their role in this scenario, it is important to consider their potential in becoming pro-oxidant substances, when they lose electrons or when they are reducing agents [111,119]. Excessive production of ROS can be associated with the development of metabolic disorders and inflammation [133]. Therefore, it is important to combat this imbalance and treat the root cause to prevent further health implications. Polyphenols can modulate the immune system in NOD-like receptors, Toll-like receptors, NF-ĸB, proinflammatory chemokines, cytokines, and adhesion molecules, because of the existence of 4–5 hydroxyl groups in their molecule, resulting in antioxidant potential [121].

#### 3.3.4. Anticancer Activity

A cancerous cell is defined as a life unit that is prone to proliferation, motivated by the genetic history of the individual or other habitual or environmental factors (diet, lifestyle, pollution, radiation, and many others). In 35% of cancer cases, the causes are dietary habits and patterns [134]. Since phytochemicals like polyphenols are great anticancer agents, after being studied for their properties, they could be used as support for cancer treatment [135]. With their affinity for protein macromolecules, polyphenols exert benefits in the field of interest of cellular effects.

Mainly, they are used as an aid in chemotherapy side effects, or preventive tool to ameliorate chemotherapeutics. In animal models, they show inhibition of cell proliferation, differentiation, apoptosis, angiogenesis, or metastasis. Moreover, they can reduce the possibility of different types of cancers to develop [119,134]. As anticancerogenics, some polyphenols demonstrate important effects on certain types of cancers. Quercetin and green tea catechins can be inhibitors for the tumor’s activity in colon cancer and protection from different GI cancers [134], supposedly having a local antibiotic effect. Injection with resveratrol in breast cancer reduces the carcinogenic activity of immunosuppressor dimethylbenz[α]anthracene, used in animal studies. Genistein also has successful attribution for mammary cancer [134].

Polyphenols are responsible for the modulation of cytokine-signaling pathways that involve cancerogenic cells. Green tea polyphenols, especially Epigallocatechin-3-gallate and other tea catechins, are important for their antioxidant, anti-inflammatory, and anticancer capacities due to the multitude of OH groups [121]. Chung S. Yang et al. demonstrated the effectiveness of tea catechins in suppressing proliferation of cancer cells and inflammation [136]. Red wine interactions with colon cancer and tumors have been studied, and the researchers showed the success of phenols present in the beverage in carcinogenesis suppression. Polyphenols are capable of lowering DNA damage caused by ROS formation, and therefore inhibit cancerous cells and their multiplication [121].

Inhibiting angiogenesis can be used in cancer treatments as the process itself involves generating new blood vessels from already existing ones. Unregulated angiogenesis is described in diseases like psoriasis, hemangioma, tumor growth, and metastasis. Inhibiting this process could prevent these cancerogenic cells from spreading, and therefore, inhibit their way of getting to other organs in the body. Factors of this process include vascular endothelial growth (VEGFs) and basic fibroblast factor (bFGF) [137,138]. Both of them are important for the endothelial cell’s survival, differentiating, and migrating. Enzymes in collagen degradation such as matrix metalloproteins (MMPs) are secreted by endothelial cells and they are responsible for the cell’s movement, because for that to take place, it previously is in need of degradation to happen. Polyphenols can inhibit angiogenesis via direct suppressive effects. Resveratrol is used as a suppressant for malignant tumors (leukemia, neuroblastoma, breast cancer, intestine cancer, liver cancer, colon cancer). Resveratrol is known to cause apoptosis and G1/S-phase cell cycle arrest in cancer cells. It downregulates the activating of kappa B (NF-κB) and the expression of MMP-9, survivin, cyclin D1, COX-2, and intracellular adhesion molecule 1. Antiangiogenics are complex and include the downregulating of VEGFs and FGFs in endothelial cells, mediated by the reduction of hypoxia-inducible factor-1α and the suppression of thrombospondin-1 and tumor suppressor factor 53. Other compounds, such as genistein found in soybeans, can be tools for prostate, breast, gastric, and colon cancer, with anticancer mechanisms that include the inhibition of tyrosin kinase, topoisomerase 20S, proteasomal activity, and FoxM1 upregulation of p27Kip-1, IKB and Bax; as angiogenetic inhibitors, the polyphenol downregulates MMP-2, HIF-1α, and VEGFs [138]. Caffeic acid phenethyl ester, existing in propolis, diminishes the cell’s capacity for proliferation in PC-3 human prostate cancer cells through reducing the phosphorylation of p-ERK1/2 (Thr202/Tyr204), p-Akt (Ser473), p-mTOR (Ser2448, Ser24981), p-GSK3α (Ser21), and p-GSK3β (Ser9) [139]. Another study shows inhibition of tumor growth in mice with the reduction of signaling molecules from the Akt pathway [140], an important protein kinase B pathway involved in cell growth, death, and survival, regulation of glucose uptake in muscle or fat cells, or suppression of neuronal cell death; dysregulation of this pathway is associated with diseases such as cancer [141].

### 3.4. Bioaccessibility of Polyphenols

The bioaccessibility of polyphenols refers to their release from the food matrix and their subsequent modifications in the gastrointestinal (GI) tract, which affect their absorption and bioavailability. Bioavailability is influenced by factors such as the molecular structure of polyphenols, the interactions they have with other dietary components, and the mechanisms they undergo in the body. When bioavailability is studied, it is essential to first understand the resealing from the food matrix and the modifications the compound undergoes in the gastrointestinal tract. The majority of polyphenols ingested are not excreted through urine, meaning that they either have not been absorbed properly in the gut, absorbed, and excreted in the bile, or metabolized by the colon microflora or by the tissues. The molecular structure of polyphenols in their free form gives them the benefit to pass through plasmatic membranes by diffusion, unlike the case when they form complexes with other compounds, changing the way they get assimilated [111]. The availability of the compound is bound to the presence of other molecules interacting with the phenolic compound: dietary fiber reduces the absorption rate, trapping the phenols in the matrix due to their polysaccharides and the polar groups of the polyphenols; lipids increase absorption in the intestine for non-polar phenol groups (curcumin, resveratrol, xanthones, and some flavonoid aglycones); dietary proteins, on the other hand, interact with the hydroxyl groups of the polyphenols, forming hydron bonds and hydrophobic ones with the carboxyl groups of different protein chains, increasing absorption 1.5-10 times [142]; dietary carbohydrates can increase absorption of flavanols to 40%, whereas generic absorption of polyphenols is also increased due to high GI motility and action of enzymes [10,111].

#### 3.4.1. Absorption in Stomach

Mechanic action that happens to the ingested foodstuff, such as chewing, the crushing process, gastric motility, and abrasion between the aliments can ease the liberation and assimilation of polyphenols [111]. The stomach can only accept the free form of polyphenol for absorption. It has been proved that acidic pH does not affect the stability of polyphenol and that, depending on the complex form, the aglycon can be liberated [28]. If the polyphenol has an affinity to the mucus excreted when chewing, the mucins act as a protective layer, not allowing the gastric absorption to take place. The phenols or flavonoids found in the extracellular matrix could only be dissolved in alkaline conditions, but not in acid ones. However, they might provide local activity, protecting the GI tract of oxidizing agents’ exposure, inflammation, and intestinal diseases. Chlorogenic acids undergo hydrolysis, resulting in the release of caffeic acid and quinic acid [83,115]. It is important to note that, unlike flavonoid glycosides, chlorogenic acids are esters formed by the conjugation of caffeic acid and quinic acid. Therefore, their hydrolysis produces specific hydrolysates (caffeic acid and quinic acid) rather than aglycones [111,143].

The complexes polyphenols form in the stomach make it difficult to understand the GI pathway and its mechanism in human trials. In vitro studies provide an understanding of gastric uptake. In most cases, absorption of polyphenols in the stomach does not occur. Only in the case of free and conjugated Hydroxytyrosol and Tyrosol is encountered a hydrolysis of phenolic compound conjugates, according to Mireille Koudoufio and collaborators. The hydrolyzation of procyanidins occurs as a time-dependent hydrolysis of oligomers, and achieves polyphenol stability when faced with a low pH [143].

#### 3.4.2. Absorption in the Small Intestine

The first step in the intestinal process, due to sugar linkage of polyphenols, would be glycosylation by the intestinal mucosa, or by microorganisms existent in the gut. Rhamnose-linked phenols cannot be metabolized in the intestine, and therefore, will further be transported in the colon. In the duodenum, most polyphenolic compounds pass by intact, while high-molecular-weight ones are hydrolyzed into dimers or monomers by bile salts and pancreatin-stimulated dialyze and non-dialyze [143]. Whereas the other polyphenols, like aglycones and some glucosides [28], have two possible pathways for absorption in the jejunum and ileum segments. One is the transportation in intact state to enterocytes (IPEC, a strain of cells existing in human GI tract [143]) by sodium-dependent glucose transporter, followed by hydrolyzation by cytosolic β-glucosidase, resulting in their aglycone. The second one is the glucosidase of the brush border membrane, lactase phlorizin hydrolase (LPH), that catalyzes extracellular hydrolysis of some glucosides, and then diffusion of the aglycone [28,143]. Flavonoid glucosides involve both pathways: Quercetin 3-glucoside, mono-glucosides of genistein and daidzein with LPH, cytosolic β-glucosidase (CBG) for hydrolyzing Q4’G, genistein 7-*O*-glucoside (genistein), and daidzein 7-*O*-glucoside (daidzein); quercetin-4′-glucoside seems to use both [28,144].

After entering the villi (epithelial of the small intestine), conjugation occurs through either methylation, sulfation, or glucuronidation [144]. The type of conjugation is related to the dose, and the nature of substrate of the ingested compound. Sulfation takes place in cases of lower-dose high-affinity substrates, shifting towards glucuronidation when a dose increases [28]. In a study regarding the Spanish diet, it is estimated that 48% of the total polyphenols from vegetables are bioaccessible in the small intestine. A considerate number of studies show that only 5–10% of the bioaccessible polyphenols get to be metabolized through absorption in the intestinal villi [145].

#### 3.4.3. Colon Absorption

**Figure 7:**
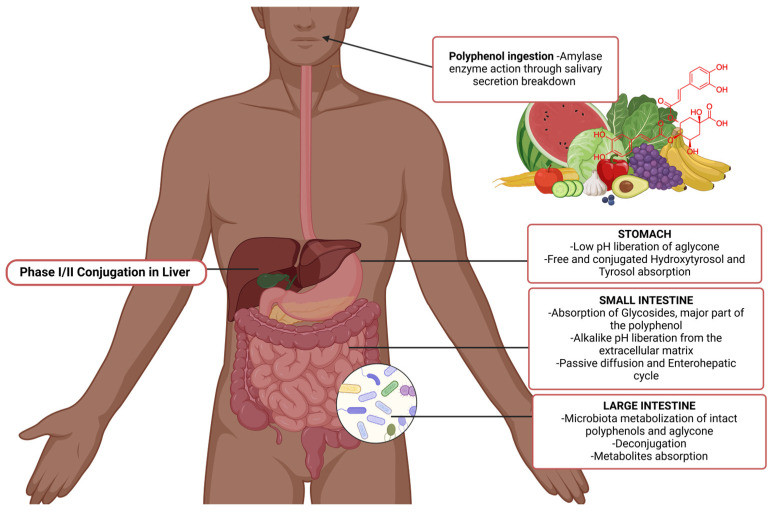
Representation of the general absorption of polyphenols.

Polyphenols that are not metabolized in the stomach or the small bowel will pass further into the colon, where they are subjects of destruction until reaching phenolic acid compounds (Figure 7).

Glycosides that arrived from the small intestine, undigested, will be transformed into aglycones by hydrolyzation with the help of the colonic microflora, catalyzed by β-glucosidase, β-rhamnosidase, or esterase. The aglycones resulted will be absorbed via the portal vein to the liver, either execrated in the bile back into the small intestine, creating an enterohepatic cycle while also creating a synergetic relationship between the gut microbiota and polyphenols [143]. In the liver, like in the intestine, after absorption, resulted fractions of polyphenols undergo the same types of conjugations (also called phase II enzymatic conversion) [144]. A possible pathway is simply by excretion into feces, along with the undigestible fraction present in plant tissue, but according to studies, only 10% of polyphenols experience this type of elimination, resulting in the utility of gut-assimilated polyphenols up to 90%. Hepatic function also implies that polyphenols can be transported to tissues or by renal pathway (phase I conversions) into urine. The interaction of microbiota and polyphenols has a great interest in the use of these phytochemicals as prebiotics. Studies conducted in vivo and in vitro show the modulation of gut metabolism and inflammatory pathway by inhibiting mast cell degranulation. They also show potential in improving gut barrier protection, and improvement in metabolic homeostasis. As a first conclusion so far, polyphenols go through changes from the moment they encounter the enzymes present in saliva. They get liberated from conjunction, re-conjugate in the liver or the intestine, get used up by microflora’s bacteria after assimilation by the GI tract, enter the composition of plasmatic proteins, and at last, incorporate in adipose tissues [111]. In vitro studies show ideal course of metabolic reactions happening in the human body, at level of digestion and possibly target-action of polyphenols towards tissues and cells, exerting beneficial effects. In vivo cases, however, cannot always be the most accurate, due to individuality. Each organism differs in one way or another, and human metabolism is harder to understand fully because of the many possible interactions and structures that form through the catabolism’s (and anabolism’s) track.

Even though in vitro studies do not give us exact data about human bioavailability of polyphenols, they help know how many beans make five, meaning that they give us an idea of the asset that are these compounds for health.

## 4. Major Polyphenol Food Sources and Their Bioavailability

Due to their consumption in a larger quantity, two food sources of major polyphenol compounds were analyzed, in hopes for a better understanding of the polyphenols’ bioavailability. Their everyday consumption leads to daily CGA and quercetin sources, each of the chosen foodstuff having a corresponding polyphenolic group in a significant amount. The impact on well-being is linked to the quantity ingested and the metabolism’s capacity of utilizing the compounds in their free form or metabolites formed throughout the digestion. For coffee and onion, we demonstrated the bioaccessibility from the moment of ingestion through the GI tract, but we also appreciated the bioactivity these compounds possess.

### 4.1. Coffee—Coffea Arabica and Coffea Robusta

Coffee, a simple infusion of ground coffee beans, as it is seen by most people. In reality, coffee is a complex antioxidant bomb, containing high amounts of polyphenolic compounds. Besides giving a boost of energy, it offers benefits regarding the neurological system, cardiovascular system, and also digestive system (and many others). Green coffee, less accepted by the population due to intense body and spicy aftertaste, confers maximum antioxidant activities and better absorption. Roasted coffee, on the other hand, is a well-liked beverage, appreciated all over the world. The downside is that in the process of roasting the beans, it loses a few of the antioxidant properties, depending on the assortment (*Arabica*, *Robusta*, *Excelsa and Liberica*), origin (country where it has been grown), preparation (brewing, boiling, filtering) also influences the polyphenolic quality and grade of roasting. Besides roasting, coffee can undergo other types of processing such as washing, introduction of carbon dioxide (Anaerobic Processing), natural drying and aerobic fermentation. In coffee, in the highest amount, is encountered chlorogenic acid (CGA). CGAs are abundant in fruit and vegetable species. The most representative sources of CGAs are coffee beans, potatoes, eggplants, and sunflower seeds. They exist displayed as four different isomers, 1-CQA, 3-CQA, 4-CQA, 5-CQA, caffeoylquinic acid. Coffee is considered to be the main source, with the largest amount of CGAs, with concentration being 6–12% [16].

Research shows the most common in coffee beans is 5-CQA, representing 76–84% of the total CGAs, an approximate value of 10 g/100 bg coffee beans, and total CGAs ranging from 1.76–88.0 mg/g. A study was conducted by Huijie Lu et al., where they interpreted the data from two other studies conducted in 1984 and 2008 [16]. Close values were obtained by Joanna Grzelczyk and collaborators, when testing samples of *Arabica* (*C. Arabica, fam. Rubiaceae*) and Robusta (*C. Canephora*, *fam. Rubiaceae*) on different roasting levels [146]. In a study focusing only on the ways in which roasting affects coffee beans, they also have given the conclusion that roasting level decreases levels of 5-CQAs and other CGAs. This means that CGAs are easily denaturized at high temperatures, the denaturation being directly proportional to the exposure time. Josiane Alessandra Vignoli et al. managed to use the HPLC method to determine the downfall of 5-CQAs, decreasing from 5.96 to 0.22 g/100 g for *C. Arabica* and from 6.19 to 0.13 g/100 g for *C. Canephora* [147].

A 2020 study by Vamanu et al. in Bucharest analyzed polyphenol content in brewed coffee, reporting notably low values: 135.07 ± 2.04 µg/mL (0.135 mg/g) for light roast and 56.67 ± 1.32 µg/mL (0.056 mg/g) for dark roast [148]. These values are significantly lower than typical CGA concentrations in brewed coffee, which range from 187.7 to 295.6 mg/100 mL for light roasts and 24.2 to 41.3 mg/100 mL for dark roasts [143]. Considering that 5-CQAs represent 76–84% of total CGAs, their expected content would be approximately 192.8 mg/100 mL (1.92 mg/g) for light roast and 26.2 mg/100 mL (0.26 mg/g) for dark roast [144]. The discrepancy might be attributed to factors such as processing methods, roasting intensity, or coffee bean quality [149,150].

In Table 5, the differences between coffee types and roasting methods can be observed; we highlight that in Robusta coffee, the quantity of 5-CQAs is higher than in *Arabica* coffee. The difference that appears in roasting, as the level of roasting increases, decreases the CGA content due to loss of carbon–carbon bond between the CGAs caused by heat exposure, a common trait of polyphenols.

While non-ingested quantity of polyphenol content is satisfactory, in the digestion phase, the situation changes. Bioavailability depends on an amalgam of intrinsic and extrinsic factors, when it is proposed discussion of in vivo digestion and absorption. In vitro, the ideal case of digestion, studies show that in the Gastric phase, concentrations of 5-CQA were low, followed by an increase in the Small Intestine phase, and another decrease in the Colonic phase, according to studies conducted by Joanna Grzelczyk et al. on Caco-2 and HT29 cells, according to roasting levels [146] and another study on digestion of coffee pulp extract conducted by Silvia Cañas and collaborators. These two studies highlight the affinity of phenolic acids for the upper part of the gastrointestinal tract, phenolic acids being characterized of it [146,151]. They are susceptive of fast and effective enterohepatic circulation, peaking at 5 min after administration.

Moreover, they are likely to “experience a rapid permeation system for intact phenolic acids and a slow permeation system for the conjugated derivatives” resulting in “conjugated phenolic acids detected in the portal vein and abdominal artery were derived from metabolism in the liver and/or re-absorption by enterohepatic circulation”, says Konishi et al. in a study conducted on rats in 2006 [152].

The study conducted by Joanna Grzelczyk et al. showed information on decreasing/increasing levels of 5-CQAs in different stages of digestion on different coffee types at various levels of roasting. Robusta green coffee managed to reach 8.24 ± 0.02 g/100 g 5-CQA in the Intestinal phase, while *Arabica* Green, the daughter of Robusta coffee, only had 2.61 ± 0.02 g/100 g 5-CQA concentration. Higher levels in all coffee types were observed at the Colonic phase, with probiotic cultures after 10 h, with a mean of 8.7 g/100 g 5-CQA. Light and dark roast concentrations decrease in all phases for both types, due to processing (roasting). An exception is the Colonic phase where it increases [146], probably because of the interaction with the gut microbiota. Joanna Grzelczyk et al. and Silvia Cañas et al. showed the same conclusion: the highest level of absorption is at the Intestinal phase [146,151]. In the case of phenolic acids, studies show that chlorogenic acids can pass through the stomach in intact form, reaching the small intestine where they undergo passive diffusion, reach the portal vein, and are further subjected to phase II conjugation [153,154] mediated by LPH by glucuronidation by UDP glucuronosyltransferase (UGT) and sulfidation by sulfotransferases (SULTs) [155]. Also, in the small intestine, CGAs with enzymatic catalysis of estrearase enzyme go through a transformation and their molecular weight lowers, thus being able to reach the liver. Transformation of CGA to caffeic acid must be transformed by colonic bacteria to m-coumaric acid and phenylpropionic derivative [153]. The Colonic phase enterprises other transformations: absorption or further metabolization, by reduction, demethylation, dihydroxylation, isomerization, and many more. Moreover, a study from 2015 by Adriana Farah and Giselle Duarte supports the statement that 90% of CGAs from coffee can be absorbed in their free form by gastrointestinal mucosa [155], due to lipophilic character, meaning that only 10% of CGAs undergo colonic phase.

**Figure 8:**
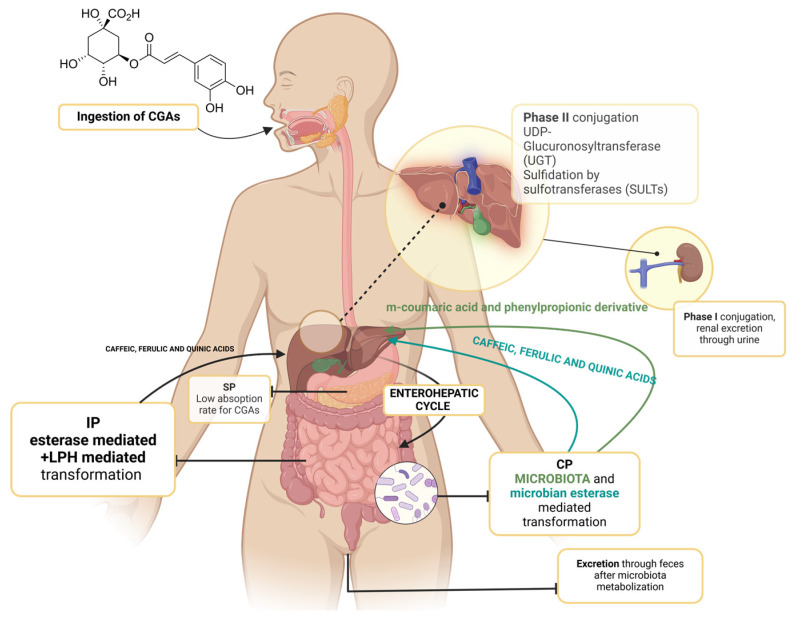
Representation of the circuit of CGAs in the GI tract. (SP—Stomach phase; IP—Intestinal phase; CP—Colonic phase).

In a study for quantification of tissue distribution and pharmacokinetics of CGAs focused on rodents, Yulu Zhou et al. showed that 5-O-caffeoylquinic acid had a rapid metabolization due to not being detected in organs after 4 h. This study also managed to indicate affinity for well-perfused organs, thus the benefits of a good operating system [156]. Excretion of chlorogenic acids can lead up to 48 h via renal pathway [157], CGAs being susceptible that they are highly implicated in the enterohepatic cycle (Figure 8).

### 4.2. Onion—Genus Allium

Most often than not, the onion is an item in the cupboard that tends to be looked down upon. Its composition proves otherwise: it is an extremely powerful one, being filled with antioxidants that fight free radicals that form in our body for different environmental influences, lifestyle choices, or diseases.

The *Allium* genus contains over 700 species, making it the largest existing category of plants [158]. It includes species such as: *A. cepa* (onion), *A. ascalonicum* (shallot), *A. sativum* (garlic), *A. schoenoprasum* L. (chives), *A. chinense* (scallion), *A. neapolitanum* (white garlic), *and A. moly* (lily leek). The focus in the following paragraphs are three species of the Allium genera, *Allium schoenoprasum*—chives, which are considered to have the strongest antioxidant power—followed by *Allium cepa*—red onion—and then *Allium cepa* L.—yellow onion [159].

Chives are plants that originated in cold regions of Europe and Asia; making an analogy to the known fact that polyphenol accumulation is linked to stress factors that are exerted on the plant, it can be a plausible reason for its highest antioxidant capacity. Chives are characterized by their thin and long leaves, with a purple inflorescence. In older times, chives were used as medicine to treat different problems like anemia, high blood pressure, act as a digestive aid, and flu treatments [159,160].

It is important to mention the existence of Sulphur compounds, as they are responsible for some biological activity of *Allium schoenoprasum*; for example, enrichment of volatile compounds. Marianna Lenkovà et al. studied the total polyphenol content (TPC) using Folin–Ciocalteu reagent and measured the Antioxidant Activity (AOA) using DPPH (2.2-diphenyl-1-picrylhydrazyl), and then, they used spectrophotometry UV/VIS at 1240–515.6 nm. The results show that the higher the number of polyphenols, the higher the antioxidant activity. Chives ranged at 1591 ± 10.89 mg$*{kg}^{-1}$ TPC and 76.57 ± 0.67% AOA [159]. Quercetin and kaempferol are two of the most important flavonoids found in the Allium family. In chives, average concentration of quercetin is at 10.4 mg/100 g fresh product, and kaempferol 12.5 g/100 g fresh product, as presented in a review conducted by Wijdan M. Dabeek and Melissa Ventura Marr in 2019 [161].

Red onions are a great source of phytochemicals that aid in human health, being anti-inflammatory, anti-obesity, antifungal, anticancerogenic, and antidiabetic. They are mostly rich in quercetin, but they are also a source of anthocyanins, giving them different nuances of red, unlike yellow onions. Yellow onions have the same benefits, to some extent, but it has been proven that red onions’ TPC and AOA are higher. In a study comparing two types of red onions, it was proven that the Sweet Italian red onion subclass had a higher level of sugar, therefore a good development of phenolic content, as we discussed about the ripening (higher sugar levels) affecting the levels of phenolic content. Rita Metrani et al. discovered, using ABTS with analysis of antioxidant activity by the power to scavenge free radicals on hydrophobic and hydrophilic constituents, that Sweet Italian had a higher AOA, while with DPPH (applicable to hydrophobic compounds) Honeysuckle had a higher activity. TPC was higher in Honeysuckle, with a possible reason that Sweet Italian had passed the certain maturity stage of maximum phenol content [162]. Marianna Lenkovà et al. reported phenolic content for the red onion variety Red Mate at 1313 ± 29.74 mg$*{kg}^{-1}$ and AOA 40.58 ± 1.157%, while for the yellow onion type Sherpa, it reached 935.2 ± 9.23 mg$*{kg}^{-1}$ and AOA 21.09 ± 2.418% [159].

In a study conducted by Jihun Lee and Alyson E. Mitchell comparing different varieties of onions, the main view upon them is comparing different layers and quercetin derivates and aglycone, measured in mg/100 g dry weight (DW). After analysis of the data provided by the study, the conclusion that the predominant quercetin-derived compound is the glycoside quercetin 4′-*O*-glucoside. In all four onions studied, it appears that high concentrations of the glucoside are found in the first and paper (skin) layer. Astounding results were achieved by the Chief variety, with 2392 ± 79 mg/100 g DW in the first layer and 1612 ± 18.5 mg/100 g DW. Satisfying results were presented for the Cowboy and Denali varieties, reaching up to 706 ± 3 mg/100 g DW in the skin, and 688 ± 11 mg/100 g DW in the first layer. Aglycones of quercetin were found in the skin for the most part, the Chief variety acquiring 423 ± 7 mg/100 g DW, while the Denali variety 326 ± 2 mg/100 g DW; Sequoia (that achieved concentrations of quercetin 3,4′-*O*-diglucoside higher comparing to quercetin 4′-*O*-glucoside) reached an aglycone concentration of 289 ± 6 mg/100 g DW. In the Denali and Cowboy varieties, aglycone concentrations were not determined [163].

These results conclude and complete the theory that environmental factors have an impact on the development of polyphenolic compounds—in this case, quercetin—with influences such as plant genetics, soil nutrients, temperature (in growth time but also storage temperature), and nitrogen concentration, according to a review conducted by Khalid Mahmud Khokhar [164]. In analogy, these factors can potentially be some of the reasons for the accumulation of phenolic content in the plants. Given that the predominant compound is quercetin, the next paragraphs explain the metabolism of quercetin.

**Figure 9:**
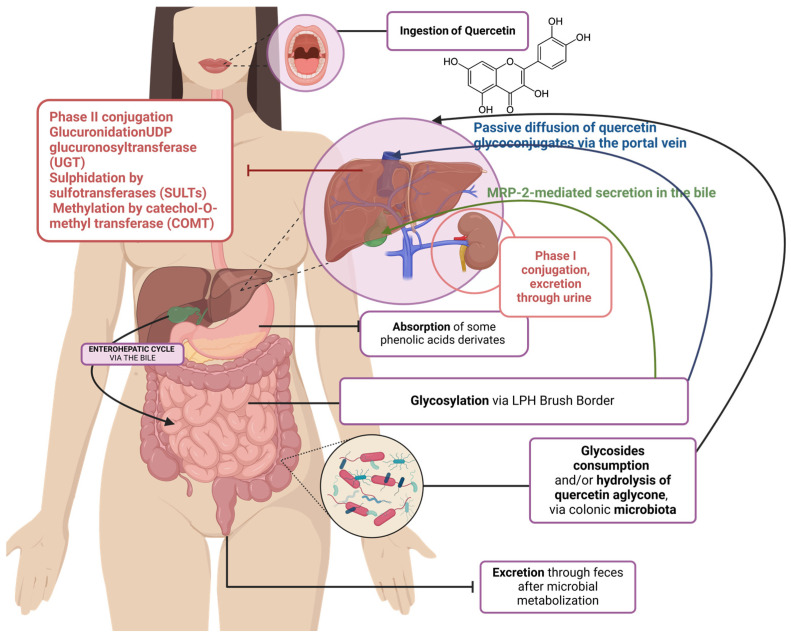
Representation of quercetin absorption.

Quercetin is hardly absorbed in the stomach, therefore transported, and primarily absorbed in the small intestine. Bondage of sugars must be removed before absorption of the quercetin aglycon. For glycosylation, LPH of the brush border removes the glucose attached, transporting it into the colon where it will undergo hydrolysis under bacteria’s action, either being completely hydrolyzed before absorption, or priorly transformed into aglycone before entering the large intestine and be absorbed by it, given the phospholipid bilayer of cellular membranes, or be transformed into aglycone by bacteria. These transformations depend on the type of sugar unit that is linked to the aglycone (Figure 9) [100]. In a study using Caco-2 cells, researchers showed that quercetin glucosides pass through the epithelial membrane easier than the aglycone, driven by the LPH, β-glucosidase hydrolysis, sodium-dependent glucose transporter1(SGLT-1) and their carbohydrate-opposing abilities to utilize the saccharides and transform them into aglycones [165]. Quercetin is degraded by gut microbiota (e.g., *Streptococcus* spp., *Lactobacillus* spp., *Pediococcus* spp., *Bifidobacterium* spp.) into phenolic acids [166].

In the intestinal lumen, they will be conjugated by glucuronidation by UDP glucuronosyltransferase (UGT), sulfidation by sulfotransferases (SULTs), and/or methylation by catechol-O-methyl transferase (COMT) present in intestinal and hepatic cells and further entering circulation. Galindo et al., in 2012, and Menendez et al., in 2011, claimed that quercetin glucuronides are more stable as phase II conjugates, but they might suffer deconjugation in vascular smooth muscle cells [167,168]. UGT-mediated glucuronidation and metabolism in the livers’ hepatocyte cells has been underlined. After passing diffusion through the portal vein, they pass through the sinusoidal membrane, and, modulated by multidrug resistance-associated protein-2 (MRP-2), arrive in the bile where they can be secreted back into the intestinal lumen [165]. The most common dietary source of quercetin is the O-glycosidic form. It includes quercetin-3-*O*-rutinoside, quercetin-3-*O*-glucoside, and quercetin-3,4′-*O*-diglucoside [161].

Intensive metabolism of quercetin is proved due to low quantities in the urine via the renal pathway; therefore, the amount of quercetin in plasma is quite high (up to 50%), as the two of them are inversely proportional [169].

For quercetin, absorption is relatively high, whilst in a slower rate, elimination half-life being of 25 h [170], the average terminal half-life (the necessary time to divide the plasma concentration by two, after the achievement of stability [171]) of quercetin is 3.5 h [172].

## 5. Methods of Improving the Bioavailability of Polyphenols

As previously mentioned, the bioavailability of polyphenols is low, and therefore, to enhance the benefits of these phytochemicals, scientists have elaborated a series of techniques for improvement of their bioavailability and delivery systems. With the creation of these delivery systems for polyphenols, their bioavailability increases, as well as their absorption in the GI tract and their organ-targeted action. Bio-based macromolecules are characterized for their affinity for molecules with a high number of hydroxyl groups, like polyphenols. The combination of these two results in increased stability and bioavailable material. Interaction with macromolecules causes the formation of these delivery systems in different ways, forming emulsions, nanoparticles, microcapsules, or liposomes [173]. Encapsulations can be emulsion-based systems or nanoparticle-based systems. Characteristic to each system is its loading capacity, encapsulation efficiency, particle size, and zeta potential. Emulsion-based systems require high-speed homogenization, ultrasonic emulsification, or other stabilizing technologies for achieving a proper desired emulsion. Liposomes can be used because of their capacity for self-assembly, modification acceptability, and compatibility [174]. Liposomes are defined as self-assembled amphiphilic spherical vesicles with at least one phospholipid bilayer, similar to the cell membrane, that is designed to separate the internal/external aqueous phase [119]. The preparation of these systems provides increased Aa activity after digestion after stimulation with intestinal fluid in the case of EGCG. In recent studies, modified liposome systems created by incorporation of the xenobiotics on the liposome surface improved the stability of the liposomes, increased the release control, and had a higher encapsulation efficiency of the tea polyphenols [174]. The importance of this method is highlighted by Minneli et al., with a success rate for encapsulation efficiency of 100% after incorporation of EGCG with Polaxomer-407 (1,2-dioleoyl-sn-glycero-3-phosphoethanolamine, 1-palmitoyl-2-oleoyl-sn-glycero-3-phosphocholine, and cholesteryl hemisuccinate). Furthermore, magnesium salt was used for maximizing EGCG internalization, obtaining in the end result a multilamellar structure through analysis with X-ray diffraction [175]. Nanoemulsions usually present themselves as a system containing an aqueous phase, oil phase, and stabilizers. They present the benefit of having a higher thermodynamic stability compared to the other emulsion types. Studies show that using hydrogel combined with water in oil emulsion increases the emulsion stability with increasing either the emulsifier used or the hydrogel, while the hydrogel shows importance in retaining the polyphenols during storing. Using oil in water emulsions is also possible, but due to the polar nature of some polyphenols (such as tea polyphenols), this process can be challenging. Pickering emulsions are stabilized by particles from protein or starch from the food matrix. They form very strong bonds between the molecule of protein/starch and the compound of interest, making it hard for them to get detached [174]. They are recognized for their high internal phase that helps them have a higher loading for polyphenols. Nonetheless, these types of emulsions can be used stabilizer-free due to the character of the bounding molecule. These systems present high ionic strength and stability against pH changes, with sizes larger than the other emulsion types, up to micron array [174,176]. Nanoparticle-based systems are made of biodegradable polymers in which polyphenols are entrapped in a particle matrix. Techniques used include spray drying, extrusion, crosslinking reactions, electrospinning, and layer-by-layer self-assembly. The nature of the polymers can be protein/carbohydrate/by-polymer-based. Protein-based systems are a good alternative thanks to their emulsification, gelatinization, and binding properties [174,177]. Milk proteins are a promising alternative, providing the formation of a complex and stable end product. EGCG-loaded casein systems have shown efficacy for their nano-structure as well as anticancer ability. Carbohydrate-based systems are biodegradable and biocompatible, forming complexes with polyphenols through non-covalent bonds, hydrogen binding, and weak ionic interactions. Starch is widely studied for its capacity for binding and forming complexes with different polyphenols. Apart from starch, alginate, inulin, cyclodextrin, and maltodextrin are other carbohydrate matrixes used in fabricating these carb-based nano-emulsions [174]. Last but not least, bi-polymeric systems are a combination of protein/peptide and polysaccharide that lead to electrostatic complexes through self-assembly. The advantages of these types of delivery systems are high encapsulation efficiency, high loading capacity, and a probable controlled release from the system [178]. They can be formed through non-covalent and covalent bonds, although a covalent bond between the protein and polysaccharide is preferred as it is demonstrated to be more stable in different environment changes and has a slower release. Covalent bonds can be obtained with chemical crosslinking agents or through Maillard reaction [174].

## 6. Toxicity of Polyphenols

Toxicity assessment of any compound advised as a treatment or adjuvant of treatment directed for human consumption is essential due to possible unwanted outcomes. The testing of compounds is required for the development of drugs and usually is achieved by testing the activity in vitro on living cells. The requirements include metabolic activity of the cells, morphology, cell growth/proliferation, or mechanisms implicated in death of the cells. Most indicated for cytotoxicity examination are human primary hepatocytes and HepG2 cell lines that provide the closest alternative to the in vivo models of the human liver. Magdalena Boncler et al. evaluated the toxicity of different polyphenols on cell cultures (HepG2, Caco-2, A549, and 3T3) using high-content screening assay. They observed that in regard to mitochondrial activity, kaempferol and four extracts of buckthorn bark, walnut husk, hollyhock flower, and silverweed herb were the most cytotoxic. The silverweed herb was one of the most toxic especially in Caco-2 cells. Membrane integrity cytotoxic activity was notably for kaempferol. In the case of nuclear area, resveratrol and kaempferol are the most concerning, in all cell lines. Omnivir R represented the most toxic compound in the nuclear area in case of HepG2 human hepatocytes. In Caco-2 cells, in comparison, Omnivir R was one of the least toxic, as well as in A549 epithelial cells. For A549 cells, Ominivir R was least toxic only in the nuclear area assay, proving information about a risk in the membrane integrity and mitochondrial membrane potential. High toxicity in these cells was represented by resveratrol and walnut husk extract. In 3T3 mouse fibroblasts, kaempferol and resveratrol possessed the highest toxicity activity in the nuclear area test. Oak bark was remarked with a high activity in all three tests [179]. Polyphenols’ capacity to chelate transition metal’s ions can be an incredible downside for people suffering with iron deficiency, as the xerobiotic can bind to iron in the intestine, thus making it impossible for Fe to be absorbed. Another effect of this interaction could lead to the dysregulation of iron homeostasis. Tea polyphenol, (-)-epigallocatechin-3-gallate, was found to be responsible for the decrease in transepithelial iron transport in Caco-2 intestinal cells. Proposed trajectory for the iron is the basolateral exit via ferroportin, which is affected by the complex formed with the polyphenol, making it unable to transport [180]. In the case of quercetin, it was observed that the complex compound formed with iron was not in the size range to be able to exit via ferropotin, and also, that the decrease in iron in Caco-2 cells was linked to a decrease in ferroportin protein and mRNA, as a mediator being miRNA with 3′UTR of ferroportin mRNA [181]. It is possible that the iron–quercetin compound formed to be retained in the enterocyte cytosol leads to iron deficiency, and is known to have an impact on the ferritin decrease. The ability of polyphenols to form complexes with proteins impairs an issue when it comes to digestive enzymes, as it could affect their normal functioning and assimilating of different food nutrients. People with food intolerances, like gluten/lactose intolerance, celiac disease exocrine pancreatic insufficiency, and complex carbohydrate intolerance, have a deficit in enzymes needed to metabolize certain food groups. Even though it is recommended for patients suffering with food intolerances to conduct a healthy lifestyle, certain polyphenols naturally found in plants (like condensed tannins, which are known to inhibit enzymes α-amylase, α-glycosidase, pepsin, trypsin, lipase, and chymotrypsin) could present an issue. Another factor to be considered is the polyphenols’ ability to stimulate and inhibit certain strains of bacteria in the microbiota. Changing the ratio in favor of non-beneficial bacteria creates dysbiosis in the gut, creating an imbalance. Dysbiosis is tightly linked to different diseases such as irritable bowel syndrome (IBS), functional dyspepsia, metabolic disorders (diabetes, obesity), inflammatory bowel diseases, colorectal cancer, and intestinal bacterial overgrowth (SIBO) [180].

## 7. Conclusions

The purpose of this study is to underline the remarkable properties of polyphenols, while also focusing on their bioavailability, as it is crucial for exerting their benefits on humans. Throughout the years, consumers have been looking for sustainable and natural medicine or treatments for diseases and thanks to the evolution of biotechnology, now they can choose from a variety of alternatives to treat or ameliorate their symptoms, conducting to a better health. Polyphenols are secondary metabolites of plants, formed in times of defense against different environmental and/or genetic factors. Hundreds of years were needed to quantify and classify them and to determine their contribution to well-being. As for their bioavailability, it is certain from the research conducted that polyphenols are quickly absorbed and assimilated, a reason for their low oral bioavailability. Most of the polyphenols are not absorbed in their free form; nevertheless, the benefits of consumption still exist, being transformed and converted into metabolites that reach the liver, kidneys, and other organs in the body, exerting different actions like antioxidant (scavenging ROS), antimicrobial, and anticancerogenic. The scavenging of ROS is incredibly important since most chronic diseases and metabolic dysbiosis are linked to oxidative stress and inflammation. Their action has also been linked to the gut–brain axis by reducing the gut microbiota imbalance, improving the neurological health of the host. Discussing elimination, it depends on their circulation, implication in the enterohepatic cycle, metabolization by microbiota, and many other unknown factors implicated in digestion that are hard to put a pin into due to the complexity of the process.

## Figures and Tables

**Table 1:** HCA-rich foods and effects on health after consumption.

Plant Name	Family	HCA	Pharmacological Activity	References
*Arachis hypogaea (sprout)*	Fabaceae	*p*-coumaric acid	High-fat-diet-induced cognitive deficits, obesity, lipopolysaccharide-induced inflammation	[17,18]
*Phaseolus vulgaris* L.	Fabaceae	*p*-coumaric acid, ferulic acid	Antioxidant and reduction of inflammatory activity	[19,20]
*C. Arabica, C. Robusta*	Rubiaceae	CGAs	Cardiovascular disease, neurological disfunctions	[21,22]
*Olea europaea* L.	Oleaceae	Sinapic acid	Obesity	[23]
*Cinnamomum cassia Presl.*	Lauraceae	Cinnamic acid	Antioxidant, antimicrobial	[24]
*Eucommia ulmoides*	Eucommia	CGAs	Neuroprotection, antidepressant	[25]
*Salvia rosmarinus*	Salvia	Rosmarinic acid	Alzheimer’s disease progression, dementia, antidepressant	[26,27]

**Table 2:** Main sources of flavonoids and their biological activities.

Flavonoids	Sources	Biological Activity	References
Flavanols	*Theobroma cacao,* *Vitis vinifera,* *Camellia sinensis*	anti-inflammatory, antiviral, antimicrobial, cancer treatment adjuvant	[33]
Flavones	*Petroselinum crispum,* *Capsicum annuum,* *Apium graveolens*	reduce oxidative stress, prevention of cardiovascular disease, lower blood pressure, cholesterol lowering, blood sugar regulating	[34,35]
Isoflavones	*Glycine max,* *Allium sativum,* *Glycine max* (L.) Merr.	weight loss adjuvant, cholesterol lowering, antibacterial, hepatoprotective	[36,37]
Flavonones	*Citrus limon,* *Citrus tangerine,* *Citrus paradisi*	gut health, anti-inflammatory, antidiabetic	[38,39]
Flavonols	*Asparagus officinalis, Brassica oleracea,* *Malus domestica*	antioxidant actions, cancer prevention, hypolipidemic properties	[40,41]
Anthocyanins	*Vaccinium corymbosum, Vaccinium macrocarpon, Solanum melongena*	cardiovascular, blood sugar regulating, neuroprotective, cancer preventive	[42,43,44]

**Table 3:** Pharmaceutical properties of quercetin-rich foods in different health afflictions.

Plant Name	Family	Pharmacological Activity	References
***Apium graveolens***	*Apiaceae*	Lowers blood pressure, anti-inflammatory, antibacterial	[47,48]
***Hypericum perforatum***	*Hypericaceae*	Mental disorders, neurological effects, antimicrobial	[49]
***Brassica oleracea var. sabellica***	*Brassicaceae*	Oxidative stress	[50]
***Brassica oleracea var. italica***	*Oleaceae*	Promotes weight loss and prevents cancer	[51,52]
***Nasturtium officinale***	*Lauraceae*	Prevents risk of colorectal cancer and breast cancer,	[53,54]
***Prunus domestica***	*Rosaceae*	hepatoprotective, antimicrobial, osteoporosis, laxative	[55,56]

**Table 4:** Overview of naringenin-type flavonoids.

Flavonoid	Sources	Scientific Name	Pharmacological Activity	References
Naringenin	Grapefruit, oranges, lemons	*Citrus paradisi,* *Citrus sinensis*	Antioxidant, anti-inflammatory, antihypertensive, antidiabetic, anti-obesity, hepatoprotective	[59,60]
Naringin	Grapefruit, sweet oranges	*Citrus paradisi,* *Citrus sinensis*	Antioxidant, cholesterol lowering, anti-inflammatory, anticancer properties	[61]
Hesperidin	Oranges, lemons, tangerines, limes	*Citrus sinensis,* *Citrus limon*	Cardioprotective, anti-inflammatory, antioxidant, antihypertensive, anti-allergic	[62]
Eriocitrin	Lemons, citrons, and sour oranges	*Citrus limon,* *Citrus medica*	Antioxidant, anti-inflammatory, liver-protective, antimicrobial	[63]
Rutin	Apples, buckwheat, citrus fruits	*Malus domestica, Fagopyrum esculentum*	Anti-inflammatory, antioxidant, anticancer, blood-vessel-protective	[32,64]
Prunin	Apricots, peaches, and plums	*Prunus armeniaca, Prunus persica*	Antioxidant, anti-inflammatory, antidiabetic, cardiovascular-protective	[65,66]

**Table 5:** Content of 5-CQAs found in coffee, ranked by roasting level.

Type of Roasting	Coffee Type, Prepared by Diverse Methods					
	5-CQA Content mg/g					
	Robusta			Arabica		
Green	96.7	49.66	60.9	61.4	45.35	59.6
Light	45.3	13.87	31.5	30.1	12.08	30.5
Dark	28.2	2.42	1.3	15.1	3.25	2.2
Reference	[146]	[16]	[147]	[146]	[16]	[147]

## Data Availability

No new data were created or analyzed in this study. Data sharing is not applicable to this article.

## Abbreviations

PA	Phenolic acids
HBA	Hydroxybenzoic acid
HCA	Hydroxycinnamic acid
CQA	Caffeoylquinic acid
SDG	Secoisolariciresinol diglucoside
ED	Enterodiol
EL	Entrolactone
CGA	Chlorogenic acid
IUPAC	International Union of Pure and Applied Chemistry
LDL	Low-density lipoprotein
Sir2	Silent information regulator 2
CVD	Cardiovascular diseases
BP	Blood pressure
GABA	γ-aminobutyric acid
ROS	Reactive oxygen species
NLR	Neutrophil to lymphocyte ratio
NADPH	Nicotinamide-adenine dinucleotide phosphate
NOD	Nucleotide oligomerization domain
NF-ĸB	Nuclear factor kappa-light-chain-enhancer of activated B cells
GI	Gastrointestinal
IPEC	Intestinal porcine enterocyte cell line
LPH	Lactase phlorizin hydrolase
CBG	Cytosolic β-glucosidase
Caco2	Immortalized cell line of human colorectal adenocarcinoma cells
HT29	Human colorectal adenocarcinoma cell line with epithelial morphology
UGT	UDP glucuronosyltransferase
SULTs	Sulfotransferases
COMT	Catechol-O-methyl transferase
AOA	Antioxidant Activity
TPC	Total polyphenol content
DW	Dry weight
MRP-2	Multidrug resistance-associated protein-2
